# A Four Carbon Organonitrate as a Significant Product of Secondary Isoprene Chemistry

**DOI:** 10.1029/2021GL097366

**Published:** 2022-05-26

**Authors:** Epameinondas Tsiligiannis, Rongrong Wu, Ben H. Lee, Christian Mark Salvador, Michael Priestley, Philip T. M. Carlsson, Sungah Kang, Anna Novelli, Luc Vereecken, Hendrik Fuchs, Alfred W. Mayhew, Jacqueline F. Hamilton, Peter M. Edwards, Juliane L. Fry, Bellamy Brownwood, Steven S. Brown, Robert J. Wild, Thomas J. Bannan, Hugh Coe, James Allan, Jason D. Surratt, Asan Bacak, Paul Artaxo, Carl Percival, Song Guo, Min Hu, Tao Wang, Thomas F. Mentel, Joel A. Thornton, Mattias Hallquist

**Affiliations:** ^1^ Department of Chemistry and Molecular Biology University of Gothenburg Gothenburg Sweden; ^2^ Institute of Energy and Climate Research, IEK‐8: Troposphere Forschungszentrum Jülich GmbH Jülich Germany; ^3^ State Key Joint Laboratory of Environmental Simulation and Pollution Control International Joint Laboratory for Regional Pollution Control Ministry of Education (IJRC) College of Environmental Sciences and Engineering Peking University Beijing China; ^4^ Department of Atmospheric Sciences University of Washington Seattle WA USA; ^5^ Now at Balik Scientist Program Department of Science and Technology – Philippine Council for Industry Energy and Emerging Technology Research and Development Taguig Philippines; ^6^ Wolfson Atmospheric Chemistry Laboratories Department of Chemistry University of York York UK; ^7^ Department of Chemistry Reed College Portland OR USA; ^8^ Now at Department of Meteorology and Air Quality Wageningen University Wageningen The Netherlands; ^9^ NOAA Chemical Sciences Laboratory Boulder CO USA; ^10^ Department of Chemistry University of Colorado Boulder CO USA; ^11^ Now at Institute for Ion and Physics University of Innsbruck Innsbruck Austria; ^12^ Centre for Atmospheric Science School of Earth and Environmental Science University of Manchester Manchester UK; ^13^ Department of Environmental Sciences and Engineering Gillings School of Global Public Health The University of North Carolina at Chapel Hill Chapel Hill NC USA; ^14^ Now at Turkish Accelerator & Radiation Laboratory Ankara University Institute of Accelerator Technologies Atmospheric and Environmental Chemistry Laboratory Gölbaşı Campus Ankara Turkey; ^15^ Institute of Physics University of Sao Paulo Sao Paulo Brazil; ^16^ Jet Propulsion Laboratory Pasadena CA USA; ^17^ Department of Civil and Environmental Engineering Hong Kong Polytechnic University Hong Kong China

**Keywords:** organonitrate, isoprene, VOC oxidation, atmospheric chamber

## Abstract

Oxidation of isoprene by nitrate radicals (NO_3_) or by hydroxyl radicals (OH) under high NO_x_ conditions forms a substantial amount of organonitrates (ONs). ONs impact NO_x_ concentrations and consequently ozone formation while also contributing to secondary organic aerosol. Here we show that the ONs with the chemical formula C_4_H_7_NO_5_ are a significant fraction of isoprene‐derived ONs, based on chamber experiments and ambient measurements from different sites around the globe. From chamber experiments we found that C_4_H_7_NO_5_ isomers contribute 5%–17% of all measured ONs formed during nighttime and constitute more than 40% of the measured ONs after further daytime oxidation. In ambient measurements C_4_H_7_NO_5_ isomers usually dominate both nighttime and daytime, implying a long residence time compared to C_5_ ONs which are removed more rapidly. We propose potential nighttime sources and secondary formation pathways, and test them using a box model with an updated isoprene oxidation scheme.

## Key Points

- The C_4_H_7_NO_5_ isomers are major secondary products of isoprene oxidation during nighttime and daytime in NO_x_‐influenced regions
- The C_4_H_7_NO_5_ isomers are multi‐generational products from several C_5_ compounds that accumulate in the atmosphere
- C_4_H_7_NO_5_ as a dominant isoprene‐derived species, can be important for the formation of ozone

## 1 Introduction

Isoprene dominates biogenic non‐methane hydrocarbon emissions, contributing around 50%, followed by monoterpenes, 15%, and sesquiterpenes, 3% (Guenther et al., 2012). Isoprene reacts mainly with hydroxyl radicals (OH), ozone (O_3_), or nitrate radicals (NO_3_) (Wennberg et al., 2018), influencing surface ozone concentrations and secondary organic aerosol (SOA) formation. SOA is a major component of submicron‐sized tropospheric aerosol (Shrivastava et al., 2017) and affects human health and the climate (Glasius & Goldstein, 2016; Hallquist et al., 2009).

The oxidation of isoprene by OH radicals under low and high NO_x_ conditions has been studied extensively (D'Ambro et al., 2017; Kleindienst et al., 2007; Kroll et al., 2005; L. Lee et al., 2014; Novelli et al., 2020; Peeters et al., 2014; Schwantes et al., 2019; Thornton et al., 2020; Wennberg et al., 2018) compared to NO_3_‐initiated oxidation (Kwan et al., 2012; Ng et al., 2008; Schwantes et al., 2015; Vereecken et al., 2021; Wennberg et al., 2018; Wu et al., 2021; Zhao et al., 2021). NO_3_ is formed during nighttime from the reaction of nitrogen dioxide (NO_2_) with ozone. Oxidation initiated by NO_3_ radicals leads to significant formation of organonitrates (ONs) which add to the ONs produced during daytime oxidation under high NO_x_ conditions (Hamilton et al., 2021; Kiendler‐Scharr et al., 2016). ONs can act both as a reservoir and as a permanent sink of NO_x_ (Kenagy et al., 2020; Kiendler‐Scharr et al., 2016), and can contribute to SOA formation (Bryant et al., 2020; Fry et al., 2018; Kiendler‐Scharr et al., 2016; Lee et al., 2016; Xu et al., 2021; Zaveri et al., 2020). Investigating isoprene‐originated ONs formation is necessary to understand isoprene's effects on atmospheric NO_x_, HO_x_ and ozone formation (Li et al., 2019; Schwantes et al., 2020; Vasquez et al., 2020).

The dominant gas phase nitrated oxidation products from isoprene + NO_3_ include compounds like isoprene nitrooxy hydroperoxides (INP) (C_5_H_9_NO_5_), dihydroxy nitrates (IDHN) (C_5_H_9_NO_5_), carbonyl nitrates (ICN) (C_5_H_7_NO_4_), hydroxy nitrates (IHN) (C_5_H_9_NO_4_), and hydroxy hydroperoxy nitrates (IHPN) (C_5_H_9_NO_6_), among others (Schwantes et al., 2015; Vereecken et al., 2021; Wennberg et al., 2018; Wu et al., 2021). These compounds have also been observed in the ambient atmosphere (Lee et al., 2016; Schwantes et al., 2015; Xu et al., 2021; Ye et al., 2021).

To provide new insights into this important nitrate‐isoprene chemistry, an extensive experimental campaign focusing on isoprene oxidation by NO_3_ was performed in the large atmospheric simulation chamber SAPHIR in 2018. In this study we focus on the formation and fate of the isomers of one of the most ubiquitously detected ONs, C_4_H_7_NO_5_, and how their atmospheric fate changes between night and daytime. The experimental findings are linked to our observations of these compounds around the globe and chemical mechanisms are proposed to support our observations.

## 2 Materials and Methods

All experimental studies and field observations in this study utilized a high resolution time‐of‐flight chemical ionization mass spectrometer (Aerodyne Research Inc., hereafter CIMS) to measure nitrated organic products, using iodide as the primary reagent ion (B. H. Lee et al., 2014). The CIMS was deployed for simulation chamber experiments within a comprehensive study on nighttime isoprene chemistry, for dedicated flow reactor studies, and at several field campaign sites providing diurnal concentration profiles of selected organic nitrates.

The comprehensive nighttime isoprene chemistry study was conducted in August 2018 in the atmospheric simulation chamber SAPHIR (Section S1 in Supporting Information S1) (Fuchs et al., 2017; Rohrer et al., 2005) at Forschungszentrum Jülich, Germany (Brownwood et al., 2021; Dewald et al., 2020; Vereecken et al., 2021; Wu et al., 2021). One goal of the campaign was to explore different oxidation regimes by applying conditions enhancing the contribution of different reaction pathways (i.e., RO_2_ + RO_2_, RO_2_ + HO_2_ or unimolecular RO_2_). Nevertheless, the dominant loss of RO_2_ were the reactions with HO_2_ or NO_3_, as detailed in a previous manuscript (Brownwood et al., 2021). Here we focus our analysis on four selected experiments: one experiment enhancing HO_2_ by propene ozonolysis (exp. 1), one favoring the RO_2_ + RO_2_ pathway (exp. 2), and two experiments in which a nighttime to daytime transition was achieved by opening the roof of the chamber after the oxidation products of isoprene and NO_3_ had accumulated (exp. 3 & 4, Table S1 in Supporting Information S1). The daytime chemistry favored either oxidation by OH in exp. 3, or only photolysis by scavenging OH by CO addition in exp. 4. Exp. 3 favored RO_2_ isomerization and also contained seed aerosol to test effects of heterogeneous chemistry, whereas exp. 4 favored the RO_2_ + RO_2_ reactions. This had minor effects on the general evolution of gas‐phase products, that is, only a small fraction of the accretion ONs would partition to the particle phase (Wu et al., 2021). Experiments took place in Gothenburg using the laminar‐flow Go:PAM reactor (Tsiligiannis et al., 2019; Watne et al., 2018) to test for a possible formation of the target compound(s) C_4_H_7_NO_5_ from NO_3_ oxidation of methyl vinyl ketone (MVK) (Table S8 in Supporting Information S1) and to constrain the instrument's sensitivity to ONs (Lopez‐Hilfiker et al., 2016) (Table S3 in Supporting Information S1). Here NO_3_ was introduced following the decomposition of N_2_O_5_ added via a diffusion source (Sections S2 and S5 in Supporting Information S1).

The ambient concentration profiles of the target isoprene products C_4_H_7_NO_5_ were characterized during six field campaigns. Two campaigns took place in Asia, in Changping (near Beijing) (Le Breton, Wang et al., 2018) and Hong Kong as part of a project on photochemical smog in China (Hallquist et al., 2016). Two additional sites were located in Europe, in Jülich, Germany, during the JULIAC campaign and in Gothenburg, Sweden. Finally, data from Alabama, in the south‐eastern USA during the Southern Oxidant and Aerosol Study (SOAS) (Lee et al., 2016) and from the Amazon rainforest were used to illustrate the omnipresence of the C_4_H_7_NO_5_ isomers (Section S3 in Supporting Information S1). The C_4_H_7_NO_5_ signal has also been observed in the free troposphere as part of flight measurements over the south‐eastern USA during the Southeast Nexus campaign. However, a detailed discussion on those flights will be presented elsewhere.

The University of Gothenburg CIMS (GU‐CIMS) was used for most field and laboratory measurements. The measurements from the SOAS campaign used the University of Washington CIMS (UW‐CIMS), while the Amazon rainforest measurements used the University of Manchester CIMS (UMan‐CIMS). Additional information on operational characteristics of CIMS during each campaign are given in Table S6 in Supporting Information S1. A bulk ON sensitivity factor of 4.8 ncps ppt^−1^, derived during the SAPHIR experiment was used to convert the measured ONs signal to concentrations by GU‐CIMS, where potential variability between ON was investigated using a voltage scanning method (see Section S2 in Supporting Information S1). UW‐CIMS used a weighted isomer distribution of the isoprene‐derived ON (C_5_H_9_NO_4_, IHN) and UMan‐CIMS used the isoprene‐derived IEPOX as a proxy calibrant. More details on calibrations, sensitivity estimations and assumptions can be found in the SI (Section S2 & S3 in Supporting Information S1).

## 3 Results and Discussions

### 3.1 Experiments in the Atmospheric Simulation Chamber SAPHIR

During the experiments in the SAPHIR chamber, 24 mononitrates, 22 dinitrates and 18 accretion products were identified using the CIMS. Mononitrates dominated the spectrum ranging from 80.7% to 96.4% of the measured ONs, followed by dinitrates (3.3%–17.9%) and accretion products (0.2%–1.5%). Formation of ions assigned to the chemical composition C_4_H_7_NO_5_ (Figure S2 in Supporting Information S1) were evident in all experiments. C_4_H_7_NO_5_ signal was identified by the CIMS as an important nitrated product(s) together with the primary products C_5_H_9_NO_5_ (hydroperoxide nitrates, INP), C_5_H_7_NO_4_ (carbonyl nitrates, ICN), and C_5_H_9_NO_4_ (hydroxy nitrates, IHN). Herein INP forms from RO_2_ + HO_2_ reaction whilst ICN and IHN largely form from RO_2_ + RO_2_ reaction.

**Figure 1:**
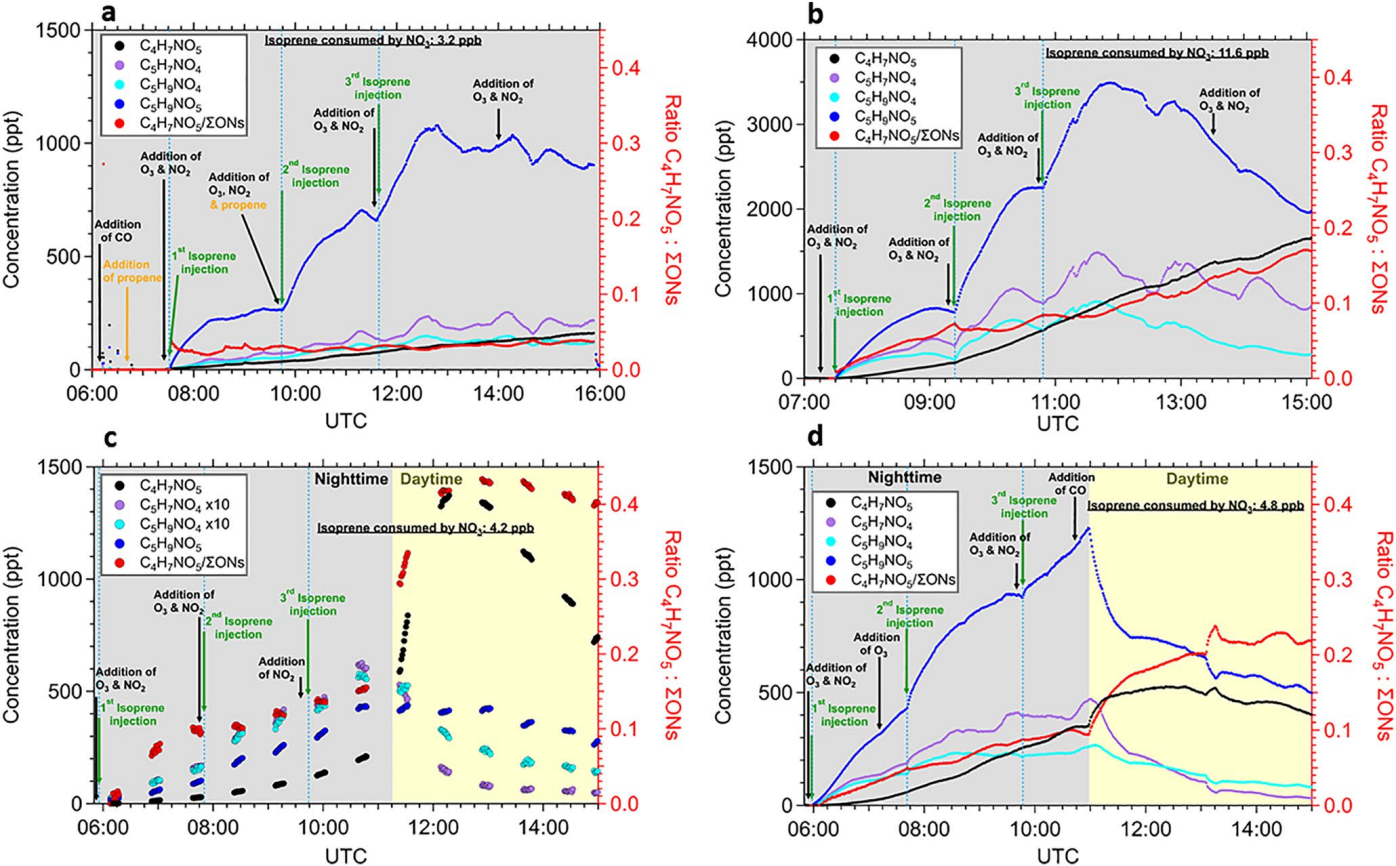
(a) Exp. 1 favoring RO_2_ + HO_2_ reactions. (b) Exp. 2 favoring RO_2_ + RO_2_ reactions. (c) Exp. 3 Nighttime‐daytime transition focusing on the effect of the OH oxidation during daytime. (d) Exp. 4 Nighttime‐daytime transition focusing on photolysis, using CO scavenger to suppress OH chemistry.

Figure 1 depicts the time evolution of C_4_H_7_NO_5_ (black), and the three other major primary oxidation products, C_5_H_9_NO_5_ (blue), C_5_H_7_NO_4_ (purple), and C_5_H_9_NO_4_ (cyan), during the four selected experiments (Table S1 in Supporting Information S1). The relative contribution (red) of C_4_H_7_NO_5_, expressed as the ratio of C_4_H_7_NO_5_ over the total measured ONs signals by CIMS is also shown. The relative contribution is estimated assuming the same sensitivity for all the measured ONs. Generally, the time‐series of the sum of measured mononitrates, dinitrates and accretion products followed the total gas‐phase alkyl nitrates time evolution measured by a Thermal Dissociation Cavity Ring‐down Spectrometer during the campaign (Brownwood et al., 2021), suggesting the CIMS total ONs signal includes the majority of the formed ON. In the dark the primary products increased rapidly after the injection of oxidant precursors and isoprene (Figure 1), especially C_5_H_9_NO_5_ (INP)_,_ the dominant primary nighttime ON measured by the CIMS in all experiments. The C_4_H_7_NO_5_ increased slowly and steadily, suggesting that there was no strong sink during the NO_3_‐dominated nighttime oxidation. This behavior would be typical for a closed shell product. However, the yield of C_4_H_7_NO_5_ strongly depended on the chemical regime. In exp. 1 (Figure 1a) RO_2_ + HO_2_ reactions dominated (91%), while the RO_2_ + RO_2_ reactions were responsible for only a 4% loss (Brownwood et al., 2021). In exp. 2 (Figure 1b), the RO_2_ + RO_2_ reactions contributed up to 13% and the RO_2_ + HO_2_ reactions 53% of the loss rate of RO_2_ (Brownwood et al., 2021). Under dominant HO_2_ conditions (exp. 1) the C_4_H_7_NO_5_ formation was lower, and its relative contribution was always less than 5%, while the C_5_H_9_NO_5_ (INP) contribution to ONs ranged between 21% and 33% due to the enhanced importance of the HO_2_ reaction for the primary peroxy radical from isoprene + NO_3_ reaction. When the RO_2_ + RO_2_ reactions were more important (exp. 2), the relative contribution of C_4_H_7_NO_5_ increased, ultimately reaching over 15% and becoming the second major remaining product with a contribution close to that of C_5_H_9_NO_5_.

Toward the end of exp. 1 and 2, when all isoprene was consumed, oxidant precursors were added without additional isoprene to enhance the oxidation of the products. For the experiment favoring RO_2_ + HO_2_ (exp. 1), this addition did not have a substantial effect on the products, including C_4_H_7_NO_5_. For the conditions where RO_2_ + RO_2_ became more important (exp. 2), the signal of major primary products decreases, thus the relative contribution of C_4_H_7_NO_5_ to the total ONs further increases from around 10% before the enhanced NO_3_ oxidation to more than 15% at the end of the experiment. Thus, the RO_2_ + RO_2_ regime enables a subsequent acceleration of secondary chemistry and multi‐generation products (Wu et al., 2021).

Nighttime to daytime transitions were included in exp. 3 and 4 by exposing the reaction mixture to sunlight by opening the roof of SAPHIR after a period of dark NO_3_ oxidation (Figures 1c and 1d). The period of NO_3_ oxidation was similar to exp. 2 without the last oxidant‐only addition. The relative contribution of C_4_H_7_NO_5_ to the total ONs was around 10% at the end of the nighttime period. Under daytime conditions in exp. 3, the ONs concentrations are expected to decrease under low NO conditions, as the carbonyl nitrates from isoprene react with OH radicals or are rapidly photolyzed (Müller et al., 2014; Xiong et al., 2016). This was also observed here for most of the ONs, for example, C_5_H_7_NO_4_ (ICN) and C_5_H_9_NO_4_ (IHN). However, the signal of the major product C_5_H_9_NO_5_ did not decrease, that is, either C_5_H_9_NO_5_ is not affected by daytime chemistry, or there are processes counteracting the loss, for example, formation of a nitrooxy hydroxyepoxide (INHE) by OH oxidation as described by Schwantes et al. (2015). The most pronounced change was the strong increase of the C_4_H_7_NO_5_ signal, which became the dominant nitrated product(s), increasing from 10% at the end of nighttime to over 40% after an hour of sunlight. At the onset of this enhanced increase of C_4_H_7_NO_5_, there was no isoprene left in the chamber, clearly demonstrating the multi‐generational sources of this product(s). In accordance with Schwantes et al. (2015) and Wennberg et al. (2018) we propose that all three major primary C_5_ ONs (C_5_H_9_NO_5_ (INP), C_5_H_7_NO_4_ (ICN), and C_5_H_9_NO_4_ (IHN)) can react with OH to form products with the chemical formula C_4_H_7_NO_5_. After one and a half hours, C_4_H_7_NO_5_ started decreasing due to chamber dilution, decreased availability of its precursors and slow but persistent removal processes, such as reaction with OH, photolysis.

To separately study the effect of photolysis, CO was added as an OH scavenger during the daytime period in exp. 4 (Figure 1d). In contrast to exp. 3, C_5_H_9_NO_5_ decreased extremely rapidly as soon as the roof was opened. This difference to the daytime period of exp. 3 supports the existence of a formation pathway of C_5_H_9_NO_5_ by OH oxidation that can balance out the significant loss by photolysis, as observed in exp. 4. In contrast, the C_4_H_7_NO_5_ signal still increased in exp. 4 but to a lesser extent than in exp. 3. From the experiments in the simulation chamber, we conclude that formation pathways of C_4_H_7_NO_5_ compound(s) exist in the dark (5%–17% of measured ONs), while during the following daytime, OH oxidation of multi‐generational NO_3_ products, together with a smaller contribution by photolysis, leads to a significant formation of C_4_H_7_NO_5_, ultimately contributing more than 40% of measured ONs.

### 3.2 Ambient Measurements

**Figure 2:**
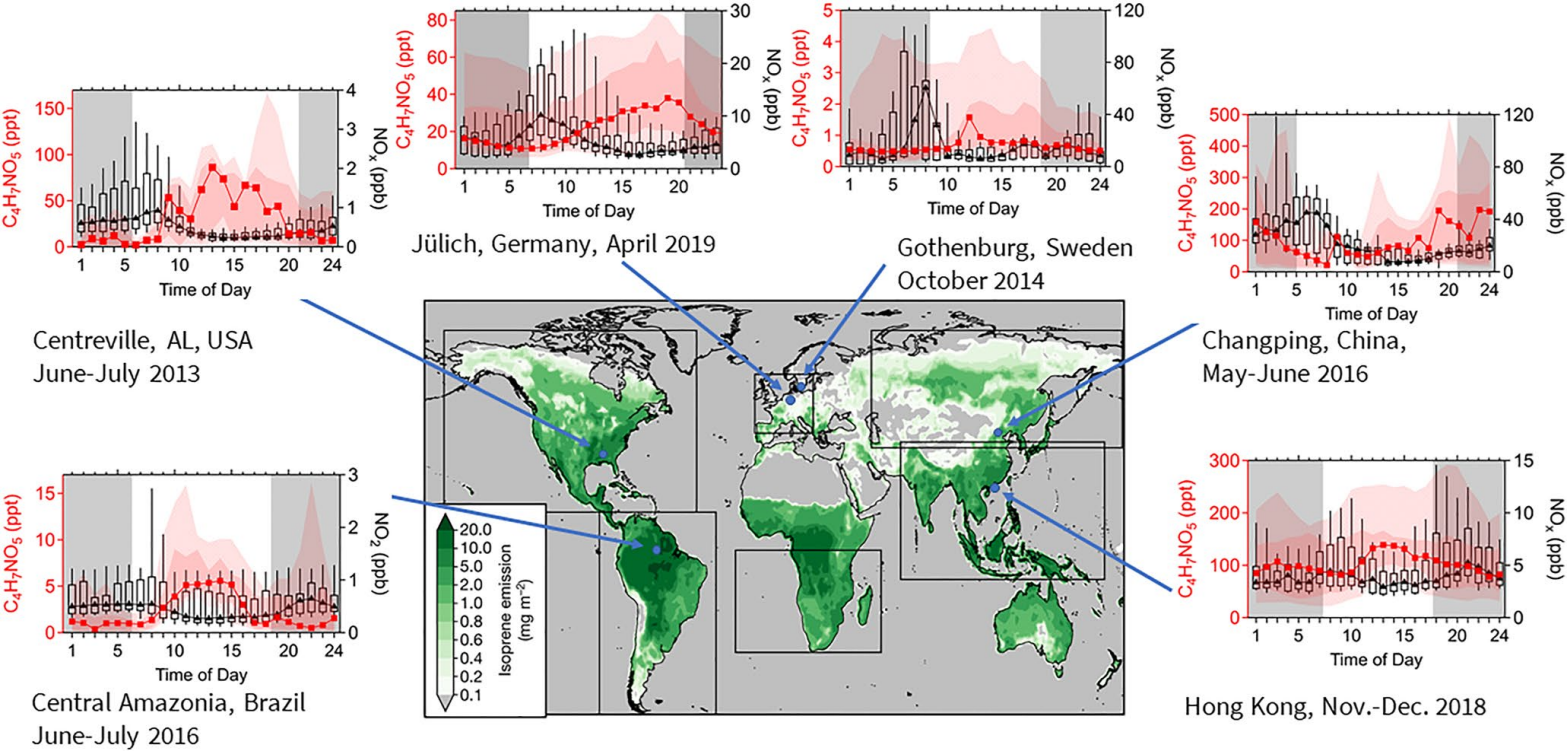
Median diurnal profile of the C_4_H_7_NO_5_ and NO_x_ in six different locations, with the 10th, 25th, 75th and 90th percentile. The gray areas in the plot indicate the nighttime period. Only the nitrogen dioxide profile is depicted in Amazonia. The map with the isoprene emissions is adapted from McFiggans et al. (2019).

C_4_H_7_NO_5_ and the other major ONs observed in the chamber experiments were measured in six different field locations around the world. Overall, C_4_H_7_NO_5_ signal was the dominant isoprene‐derived nitrate measured during both daytime and nighttime at all the sampling sites (Figure S4 in Supporting Information S1). The ratio of C_4_H_7_NO_5_ to the major primary C_5_ ONs (C_5_H_9_NO_5_, C_5_H_7_NO_4_, and C_5_H_9_NO_4_) often exhibited values above one throughout the day. However, the diurnal profile of C_4_H_7_NO_5_ differed from site to site (Figure 2).

In Hong Kong – an isoprene‐rich area with influence from anthropogenic emissions (Peng et al., 2022) – there were two peaks, one during daytime and one during nighttime. The nighttime peak becomes more prevalent if the period with high ONs formation is selected (between 14–25 November, Figure S5 in Supporting Information S1). Then, the median maximum value of the nighttime peak increases from 107 to 135 ppt and the daytime from 139 to 159 ppt. The fraction of C_4_H_7_NO_5_ in the total measured isoprene‐derived ONs ranged from 5% to 40% during the Hong Kong campaign (Figure S5 in Supporting Information S1).

The profile in Changping (near Beijing, China) showed a higher contribution during night than during day in conjunction with higher variability compared to Hong Kong. The isoprene mean diurnal profile had a peak at 14:00 and the isoprene concentrations were almost always above zero, even during nighttime (Le Breton, Hallquist et al., 2018). The local meteorology has a pronounced impact on the type of air masses reaching Beijing, for example, the wind speed has high values during the day and low values during the night (Le Breton, Wang et al., 2018).

The two locations in Europe are characterized by low regional isoprene emissions, especially Gothenburg, Sweden, and as expected the C_4_H_7_NO_5_ signal was much lower compared to the other sites. In Gothenburg, C_4_H_7_NO_5_ had a weak peak around noon, whilst there was a clear wide peak in the early evening in Jülich, Germany.

The Southeastern USA represents an area with high isoprene concentrations and low to modest NO_x_ emissions outside of urban areas. Centreville is a rural site with low average NO_x_ concentrations (Edwards et al., 2017; Lee et al., 2016). There, C_4_H_7_NO_5_ had a diurnal profile with a clear peak during daytime with high variability. The strong increase during morning (8:00–10:00, local time) was likely due to sampling of the residual layer after the morning breakup of the nocturnal boundary layer, enabling both production and downward transport to contribute to increasing C_4_H_7_NO_5_.

Finally, the Amazon rainforest measurements showed a strong daytime peak. The site is remote from human sources, exhibiting very low NO_x_ concentrations (average = 0.62 ppb), and consequently lower C_4_H_7_NO_5_ compared to the other isoprene‐rich areas, Hong Kong with much higher NO_x_ (average = 5 ppb) and southeastern USA with slightly higher NO_x_ levels (average = 0.67 ppb). The corresponding ozone concentrations at the measurement sites are given in SI (Table S5 in Supporting Information S1).

The correlations of C_4_H_7_NO_5_ with the other major primary isoprene‐derived ONs (C_5_H_9_NO_5_, C_5_H_7_NO_4_, and C_5_H_9_NO_4_) varied at the different sites (Table S4 in Supporting Information S1). The isoprene concentration, the origin and chemical age of the air masses, and meteorology all influenced the correlation slope and the correlation coefficient R^2^. For example, in Gothenburg local isoprene emissions are low and the correlation was likely driven by variability in air mass origin, with primary and multi‐generational isoprene products like C_4_H_7_NO_5_ exhibiting high correlation. In contrast, in areas with high isoprene emissions, such as the Amazon rainforest, the correlation between first and multi‐generational products was significantly weaker, illustrating the influence of air mass aging and the expected sequential production from isoprene.

Based on the chamber experiments, C_4_H_7_NO_5_ was the most abundant isoprene‐derived ON measured by the CIMS during daytime, but not during nighttime. The ambient measurements also showed C_4_H_7_NO_5_ was the dominant isoprene‐derived ON during daytime. However, at the ambient measurements C_4_H_7_NO_5_ was also the highest isoprene‐derived measured ON signal during nighttime. The dominant role for C_4_H_7_NO_5_ observed during nighttime could be due to efficient accumulation. Under daytime ambient conditions C_4_H_7_NO_5_ can be also produced in relatively high amounts via OH oxidation of other isoprene‐nitrated products (C_5_H_9_NO_5_ (INP), C_5_H_7_NO_4_ (ICN), and C_5_H_9_NO_4_ (IHN)). The chamber experiments showed that C_4_H_7_NO_5_ did not have any major losses by NO_3_ and O_3_. C_4_H_7_NO_5_ was also not affected drastically by the additions of isoprene or extra non‐OH oxidant during the experiments. The lack of a carbon‐carbon double bond gives C_4_H_7_NO_5_ a low reactivity toward the NO_3_ and O_3_ oxidants dominant during the night. This produces longer residence times than other major ONs that enhance accumulation of the produced C_4_H_7_NO_5_.

### 3.3 Formation and Accumulation of C_4_ Compounds

A key feature from the experiments in the chamber was the pronounced increase in concentration of C_4_H_7_NO_5_ during transitions from night to day conditions. At some of the ambient sites there was also such a tendency, but it was often concealed by the overwhelming contribution of the daytime oxidation of freshly emitted isoprene, especially for the sites with large isoprene emissions (Amazon rainforest, SE‐USA, and Hong Kong). A major result from our work is the demonstration that C_4_H_7_NO_5_ isomers are multi‐generational products of several C_5_ compounds. In the nighttime‐daytime transition this was evident both with and without OH radical chemistry (Figures 1c and 1d).

During daytime oxidation, C_4_H_7_NO_5_ isomers are expected to form by OH‐initiated oxidation via a number of pathways. For example, two major unsaturated products from OH‐initiated isoprene oxidation are MVK and methacrolein (MACR). OH addition to these compounds, followed by O_2_ addition to form a peroxy radical and subsequent NO reaction to alkoxy radicals, leads to the formation of nitrooxy ketones and nitrooxy aldehydes, with the chemical formula C_4_H_7_NO_5_ (Jenkin et al., 2015; Praske et al., 2015). OH oxidation of C_5_ ONs (C_5_H_9_NO_5_ (INP), C_5_H_7_NO_4_ (ICN), and C_5_H_9_NO_4_ (IHN)) can also lead to C_4_H_7_NO_5_ compounds (Schwantes et al., 2015; Wennberg et al., 2018). The initial peroxy radicals from the aforementioned C_5_ ONs also form alkoxy radicals from reaction with NO, or HO_2_ where a fraction can decompose to C_4_H_7_NO_5_ isomers (Novelli et al., 2021; Vereecken & Peeters, 2009; Wennberg et al., 2018).

In contrast to the daytime formation of C_4_H_7_NO_5_, where several pathways from different precursors have been suggested, little is known about nighttime formation. The most plausible reactions forming a C_4_H_7_NO_5_ isomer are listed in the supplemental and are summarized here.

(R1) $$\text{MVK}+{\text{NO}}_{3}\to \text{⋅⋅⋅}\to C_{4}H_{7}{\text{NO}}_{5}\;\left(\text{ketone}\right)\;$$

(R2) $$\text{MACR}+{\text{NO}}_{3}\to \text{⋅⋅⋅}\to C_{4}H_{7}{\text{NO}}_{5}\;\left(\text{aldehyde}\right)\;$$

(R3) $$4,1-\text{IHN}\;\left(C_{5}H_{9}{\text{NO}}_{4}\right)+{\text{NO}}_{3}\to \text{⋅⋅⋅}\to C_{4}H_{7}{\text{NO}}_{5}\;\left(\text{ketone}\right)\;$$

(R4) $$1,2-\text{IHN}\;\left(C_{5}H_{9}{\text{NO}}_{4}\right)+{\text{NO}}_{3}\to \text{⋅⋅⋅}\to C_{4}H_{7}{\text{NO}}_{5}\;\left(\text{aldehyde}\right)\;$$

(R5) $$4,3-\text{IHN}\;\left(C_{5}H_{9}{\text{NO}}_{4}\right)+{\text{NO}}_{3}\to \text{⋅⋅⋅}\to C_{4}H_{7}{\text{NO}}_{5}\;\left(\text{ketone}\right)\;$$

(R6) $$\text{HC}4\text{CCHO}\;\left(C_{5}H_{8}O_{2}\right)+{\text{NO}}_{3}\to \text{⋅⋅⋅}\to C_{4}H_{7}{\text{NO}}_{5}\;\left(\text{ketone}\right)\;$$

(R7) $$\text{HC}4\text{ACHO}\;\left(C_{5}H_{8}O_{2}\right)+{\text{NO}}_{3}\to \text{⋅⋅⋅}\to C_{4}H_{7}{\text{NO}}_{5}\;\left(\text{aldehyde}\right)\;$$

(R8) $$4,1-\text{ICN}-{\text{RO}}_{2}\;\left(C_{5}H_{8}{\text{NO}}_{7}\right)\to \text{⋅⋅⋅}\overset{\text{Decomposition}}{\to }C_{4}H_{7}{\text{NO}}_{5}\;\left(\text{aldehyde}\right)\;$$

(R9) $$1,4-\text{ICN}-{\text{RO}}_{2}\;\left(C_{5}H_{8}{\text{NO}}_{7}\right)\to \text{⋅⋅⋅}\overset{\text{Decomposition}}{\to }C_{4}H_{7}{\text{NO}}_{5}\;\left(\text{ketone}\right)\;$$

(R10) $$\text{Nitrated}\;\text{peroxy}\;\text{acids}\to \text{⋅⋅⋅}\overset{\text{Decomposition}}{\to }C_{4}H_{7}{\text{NO}}_{5}\;$$

(R11) $$\text{Nitrated}\;\text{epoxides}\to \text{⋅⋅⋅}\overset{\text{Decomposition}}{\to }C_{4}H_{7}{\text{NO}}_{5}\;$$

Although MVK and MACR oxidation by NO_3_ radicals is slow (Kwok et al., 1996) these major products could provide a persistent source of C_4_H_7_NO_5_ (Reactions R1 and R2). To verify this, further experiments were performed in an oxidation flow reactor, the Go:PAM (Table S8 in Supporting Information S1) showing a direct source of C_4_H_7_NO_5_ from NO_3_‐initiated MVK oxidation (Figure S10 in Supporting Information S1). However, the estimated maximum contribution from this pathway is very low and cannot explain the observed formation in the SAPHIR chamber.

Formation of C_4_H_7_NO_5_ during nighttime may also be due to the NO_3_‐initiated oxidation of first‐generation hydroxy nitrate isomers (IHN, C_5_H_9_NO_4_) (Reactions (R3), (R4) and R5). Here C_4_H_7_NO_5_ isomers are formed via decomposition of produced alkoxy radicals, with the specific pathway depending on the isomer. The structural differences of the IHN‐isomers also affect their rate constants with NO_3_ radicals, spanning an order of magnitude (Pfrang et al., 2006; Wennberg et al., 2018). These pathways release NO_2_ back to the system, but such an increase of NO_2_ was not observed in the SAPHIR chamber experiments. Also, structure‐activity relationship (SAR) estimates that the decomposition channel is not dominant (Novelli et al., 2021). Thus, these reactions are likely insignificant.

Another potential pathway is the further oxidation of hydroxy carbonyls (HC4CCHO and HC4ACHO, C_5_H_8_O_2_) by NO_3_ (Reactions R6 and R7). The peroxy radicals formed from oxidation of C_5_H_8_O_2_ can undergo isomerization and decomposition leading to the formation of a C_4_H_7_NO_5_ nitrooxy ketone or nitrooxy aldehyde (Figure S6 in Supporting Information S1) (Wu et al., 2021). The peroxy radicals C_5_H_8_NO_7_ can also form a C_4_H_7_NO_5_ nitrooxy carbonyl by decomposing (Reactions R8 and R9) (Wennberg et al., 2018). Those parent peroxy radicals (C_5_H_8_NO_7_) can be formed either by further autoxidation of the initial peroxy radical formed by NO_3_ oxidation (i.e., isomerization and O_2_ addition) or by OH‐initiated oxidation of C_5_H_7_NO_4_ isomers. Finally, peroxy acids and epoxides formed by the NO_3_‐initiated oxidation of isoprene (Vereecken et al., 2021) may decompose forming C_4_H_7_NO_5_ isomers (Reactions R10 and R11). However, this chemistry is not well‐known and needs further attention.

**Figure 3:**
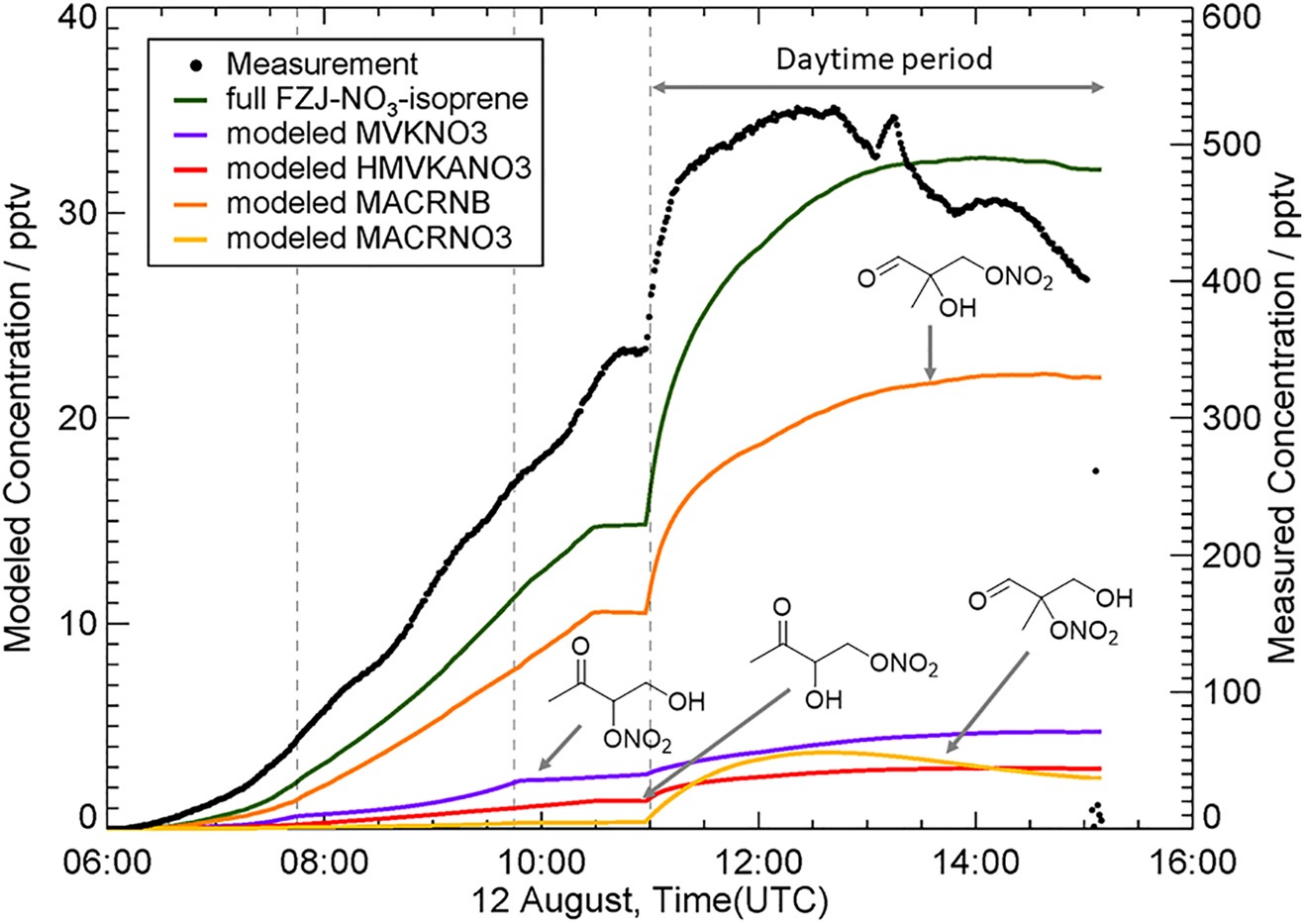
Comparison of the measured (black) and modeled (green) C_4_H_7_NO_5_ formation during exp. 4 (nighttime to daytime transition using an OH scavenger). The “full FZJ‐NO_3_‐isoprene” sum of the four main isomers is compared against the I‐CIMS measurements.

To test potential contributions of the suggested pathways, the FZJ‐NO_3_‐Isoprene mechanism presented by Vereecken et al. (2021), which in turn is based on the MCMv3.3.1 (Jenkin et al., 1997; Jenkin et al., 2015; Saunders et al., 2003) and on the more explicit descriptions from the CalTech mechanism (Wennberg et al., 2018) was expanded by the additional pathways as described above and summarized in the SI (Section S4 in Supporting Information S1). The model considered Reactions (R6), (R7), (R8), (R9). Figure 3 shows the calculated trends of four selected isomers (i.e., MACRNB, MVKNO3, HMVKANO3 and MACRNO3, with the corresponding structures shown, see also Table S7 in Supporting Information S1) for the nighttime to daytime transition experiment in the SAPHIR chamber (exp. 4, Figure 1d) with OH scavenger, where only photolysis was important. The model predicts that the dominant C_4_H_7_NO_5_ nitrooxy isomer under nighttime conditions should be the aldehyde MACRNB followed by MVKNO3, HMVKANO3 and MACRNO3 in lower concentrations (Figure 3). The overall trend of the model matches the behavior of the measurements. However, there remains a significant discrepancy in the absolute concentrations (see Section S4, Figures S7–S9 in Supporting Information S1). It is not clear why there is a such large discrepancy and as outlined in the SI some measurement concerns are addressed. Still, the general measurement agreements with for example, the total alkyl nitrates (Brownwood et al., 2021) and Br‐CIMS measurements (Section S2 in Supporting Information S1) clearly illustrate that the C_4_H_7_NO_5_ isomers are important products. This discrepancy could be due to unknown formation pathways for example, secondary formation of C_4_H_7_NO_5_ isomers from decomposition of epoxides and peroxides, highlighted as major contributing species in the model. It is not clear if produced epoxides and peroxides are unstable and can decompose into C_4_ products (C_4_H_7_NO_5_ being one of them) in the gas phase or on available surfaces, thus explaining why only low concentration of these were observed. Finally, the possibility of some production by OH oxidation under dark conditions cannot be ruled out, but certainly the contribution was small as isoprene loss due to NO_3_ was calculated to be around 90% (Brownwood et al., 2021) and the impact of isoprene + OH on the total yield of C_4_H_7_NO_5_ was below 5% for all modeled experiments (more details in Section S1 in Supporting Information S1). However, further exploration and evaluation of these pathways are beyond the scope of this work.

In the model, the increase of C_4_H_7_NO_5_ under the OH scavenged/photolytic conditions is due to the formation of the nitrooxy aldehydes MACRNO3 and MACRNB. The photolysis of two hydroperoxy aldehydes (HPALD, with chemical formula C_5_H_8_O_3_), formed via isomerization of isoprene hydroxy peroxy radicals, can lead to C_4_H_7_NO_5_ after a subsequent NO_2_ addition (Wennberg et al., 2018). One isomer forms the nitrooxy aldehyde MACRNO3 while the other forms the nitrooxy ketone MVKNO3. However, these pathways do not represent the major loss of HPALDs (Wennberg et al., 2018). Xiong et al. (2016) have suggested that C_5_H_7_NO_4_ (ICN) can dissociate via photolysis and then react with O_2_ and HO_2_ to form a vinyl hydroperoxide with chemical formula C_4_H_7_NO_5_. Since no mechanistic description was given, in this work the photolysis was implemented as given in Wennberg et al. (2018). Müller et al. (2014) have suggested that photolysis is the dominant sink of the isoprene‐derived carbonyl nitrates such as MACRNO3, MVKNO3 and HMVKANO3 under atmospheric relevant conditions, but in the model, rapid formation from HPALD and other sources counteracts their loss, leading to constant or increasing concentrations.

In addition to photolysis, the residual nighttime primary products can also react with OH radicals during daytime. We attribute the rapid C_4_H_7_NO_5_ formation in exp. 3 (Figure 1c) to the OH‐initiated oxidation of the three other major ONs (C_5_H_9_NO_5_, C_5_H_7_NO_4_, and C_5_H_9_NO_4_), which has also been proposed in previous studies (Wennberg et al., 2018) (Section S4 in Supporting Information S1). The efficiency of these pathways in forming C_4_H_7_NO_5_ must be high in order to fully explain the observations in both the findings in the SAPHIR chamber and the field observations.

## 4 Atmospheric Implication and Conclusion

The C_4_H_7_NO_5_ isomers are important products of isoprene oxidation in NO_x_‐influenced regions. Ambient measurements showed that C_4_H_7_NO_5_ compound(s) typically have higher concentrations than the other three major ONs (C_5_H_9_NO_5_ (INP), C_5_H_7_NO_4_ (ICN), and C_5_H_9_NO_4_ (IHN)) during both night and day. For nighttime conditions this finding appears in contradiction to our chamber measurements, where C_4_H_7_NO_5_ was only dominant during daytime. We found that C_4_H_7_NO_5_ isomers are multi‐generation products, with no remaining C=C bonds, in the isoprene degradation mechanism, formed from both OH and NO_3_‐radical initiated oxidation where observations in ambient air can be expected from air mass aging processes.

C_4_H_7_NO_5_ nighttime production was investigated here in detail. We suggest that the decomposition of the C_5_H_8_NO_7_ peroxy radicals from NO_3_‐initiated chemistry, the oxidation of hydroxy carbonyls (HC4CCHO and HC4ACHO) (C_5_H_8_O_2_) by NO_3_ and the decomposition of nitrated epoxides and peroxides are mainly responsible for nighttime production. The relative contribution of C_4_H_7_NO_5_ to total measured ONs increased in chamber experiments when RO_2_ loss was enhanced by RO_2_ + RO_2_ reactions. Furthermore, the chamber experiments showed that C_4_H_7_NO_5_ formation was lower during nighttime when RO_2_ + HO_2_ reactions were dominant. According to model calculations, the isomers MACRNB and MVKNO3 have the highest contribution to C_4_H_7_NO_5_ formation under dark conditions. Although most of the other ONs, generated initially in higher yields, react away after transition into the daytime, C_4_H_7_NO_5_ concentration increased, indicating a slower reactivity together with continuing or enhanced production.

The lack of a carbon‐carbon double bond lowers its reactivity and thus increases its lifetime. Slow oxidation and photolytic reactions of C_4_H_7_NO_5_ lead to longer lifetimes than those of the C_5_ ONs formed in higher yields from isoprene. This can explain the higher effective concentrations of C_4_H_7_NO_5_ in the residual boundary layer and dominance in various ambient conditions. This suggests that C_4_H_7_NO_5_ isoprene ONs could be important as a long‐term organic reservoir species of NO_x_, in comparison to the other more reactive isoprene‐derived ONs. The importance of further understanding the properties of the different isomers is highlighted by a recent study (Vasquez et al., 2020) which showed that the isoprene nitrate isomer 1,2‐IHN can efficiently remove NO_x_ from the atmosphere, whereas other isomers cannot. To further understand the distribution of isomers and their specific chemistry, further studies are needed using a broader range of methods. Especially a focus on the predicted dominant product family of the nitrated epoxides (Vereecken et al., 2021), whose secondary chemistry and therefore potential for forming C_4_H_7_NO_5_ is largely unknown, is a necessity. Finally, the isoprene‐derived ONs, of which C_4_H_7_NO_5_ is a dominant species, can thus be important for the formation of ozone, with ONs formed during nighttime affecting the initiation of tropospheric ozone formation during the following day.

## Conflict of Interest

The authors declare no conflicts of interest relevant to this study.

## Supporting information

Supporting Information S1

## Data Availability

The data used in this study are permanently archived at https://doi.org/10.5878/wfv9-a491.
