# Supplementary material for: A Four Carbon Organonitrate as a Significant Product of Secondary Isoprene Chemistry
Source: Geophys Res Lett. 2022 May 26;49(11):e2021GL097366. doi: 10.1029/2021GL097366 (PMC9285747; doi:10.1029/2021GL097366)
Supplement: Supplementary file 1 — Supporting Information S1 [file GRL-49-0-s001.pdf]

AGU PUBLICATIONS

Geophysical Research Letters

Supporting Information for

A four carbon organonitrate as a significant product of secondary isoprene chemistry

Epameinondas Tsiligiannis[1], Rongrong Wu[2,3], Ben H. Lee[4], Christian Mark Garcia Salvador[1,a], Michael Priestley[1], Philip T.M. Carlsson[2], Sungah Kang[2], Anna Novelli[2], Luc Vereecken[2], Hendrik Fuchs[2], Alfred W. Mayhew[5], Jacqueline F. Hamilton[5], Peter M. Edwards[5], Juliane L. Fry[6,b], Bellamy Brownwood[6], Steven S. Brown[7,8], Robert J. Wild[7,c], Thomas J. Bannan[9], Hugh Coe[9], James Allan[9], Jason D. Surratt[10], Asan Bacak[9,d], Paul Artaxo[11], Carl Percival[12], Song Guo[3], Min Hu[3], Tao Wang[13], Thomas F. Mentel[2], Joel A. Thornton[4,*], and Mattias Hallquist[1,*]

[1]Department of Chemistry and Molecular Biology, University of Gothenburg, Gothenburg, Sweden
[2]Institute of Energy and Climate Research, Troposphere (IEK-8), Forschungszentrum Jülich GmbH, 52428 Jülich, Germany
[3]State Key Joint Laboratory of Environmental Simulation and Pollution Control, International Joint Laboratory for Regional Pollution Control, Ministry of Education (IJRC), College of Environmental Sciences and Engineering, Peking University, Beijing 100871, China
[4]Department of Atmospheric Sciences, University of Washington, Seattle, WA 98195, USA
[5]Wolfson Atmospheric Chemistry Laboratories, Department of Chemistry, University of York, York, YO10 5DD, UK
[6]Department of Chemistry, Reed College, Portland, OR 97202, USA
[7]NOAA Chemical Sciences Laboratory, Boulder, CO 80305, USA
[8]Department of Chemistry, University of Colorado, Boulder, CO 80309, USA
[9]Centre for Atmospheric Science, School of Earth and Environmental Science, University of Manchester, Manchester, M13 9PL, UK
[10]Department of Environmental Sciences and Engineering, Gillings School of Global Public Health, The University of North Carolina at Chapel Hill, Chapel Hill, North Carolina, USA
[11]Institute of Physics, University of Sao Paulo, Sao Paulo, CEP 05507-090, Brazil
[12]Jet Propulsion Laboratory, Pasadena, CA 91109; USA
[13]Department of Civil and Environmental Engineering, Hong Kong Polytechnic University, Hong Kong 999077, China
[a]now at: Balik Scientist Program, Department of Science and Technology – Philippine Council for Industry, Energy and Emerging Technology Research and Development, Bicutan, Taguig, 1630, Philippines
[b]now at: Department of Meteorology and Air Quality, Wageningen University, Wageningen, The Netherlands
[c]now at: Institute for Ion and Applied Physics, University of Innsbruck, Innsbruck, Austria
[d]now at: Turkish Accelerator & Radiation Laboratory, Ankara University Institute of Accelerator Technologies, Atmospheric and Environmental Chemistry Laboratory, Gölbaşı Campus, Ankara, Turkey

**Correspondence to:* Mattias Hallquist (hallq@chem.gu.se) and Joel A. Thornton (thornton@atmos.washington.edu)

Contents of this file

Text S1 to S5
Figures S1 to S10
Tables S1 to S8

Introduction

Details on the isoprene oxidation experiments in the atmospheric simulation chamber SAPHIR are provided in section S1. Sensitivities for the quantification of the organonitrates are discussed in section S2. Section S3 provides details on the ambient measurements. Section S4 discusses the updated chemical mechanism of the $C_4H_7NO_5$. The last section, S5, gives details on the complimentary experiments at the Go:PAM flow reactor.

Text S1. Experiments in the atmospheric simulation chamber SAPHIR

The experiments were conducted in the atmospheric simulation chamber SAPHIR (Rohrer et al., 2005;Fuchs et al., 2017) in Jülich, Germany, in August 2018 to improve our knowledge on the gas and particle phase products of isoprene oxidation by NO_3 radicals (Dewald et al., 2020;Brownwood et al., 2021;Wu et al., 2021;Vereecken et al., 2021). Here we selected four experiments to scrutinize the formation of ONs (Table S1). The major loss of peroxy radicals was the reaction with HO_2. However, different chemical conditions enhanced different chemical regimes (Brownwood et al., 2021). In experiment 1 HO_2 formation was enhanced by propene ozonolysis and CO addition to favor the RO_2 + HO_2 regime, whereas experiment 2 favored the RO_2 + RO_2 regime. In the other two experiments we simulated nighttime to daytime transition exposing the nighttime products to OH oxidation and photolysis in exp. 3 and only to photolysis in exp. 4 where CO was added as an OH scavenger before the opening of chamber's roof. For all experiments the NO_3 radicals were formed in the chamber by the addition of ozone and NO_2. The reaction was initiated by isoprene injection. 1.5 to 2 hours after the first injection when most of the isoprene was consumed, additional NO_2, O_3, and isoprene were introduced in the chamber to propagate the chemistry. This injection of isoprene was repeated one more time in all 4 experiments (Figure 1, Table S1). In a last step, after total isoprene consumption, only ozone and NO_2 were added to enhance further oxidation of the products (exp. 1 and exp. 2). In exp. 3 and 4 the chamber roof was open to test the daytime effect on the products after the third injection. The potential aerosol contributions of isoprene products were scrutinized by addition of ammonium sulfate as aerosol seeds in exp. 3. However, the focus on the present study is on the gas-phase processes where a high-resolution time-of-flight chemical ionization mass spectrometer (HR-ToF-CIMS, Aerodyne Research Inc.) (hereafter I-CIMS) using iodide as the primary reagent ion (Lee et al., 2014) was used to measure the gas-phase oxidation products. A filter inlet for gases and aerosols (FIGAERO) (Lopez-Hilfiker et al., 2014) was also coupled to the I-CIMS during the experiment with aerosol seeds to measure the particle phase oxidation products. For clarity, the particle data was removed from the time trends shown in Figure 1c. The I-CIMS was placed in an air-conditioned container under the chamber. A four-meter long PFA (Swagelok, 6mm diameter) line and four-meter long copper tubing (12mm diameter) were used as gas and particle inlets respectively. Both were insulated to avoid condensation in the lines. The measured signal of the 64 identified ONs was corrected for background, normalized to iodide signal and converted to ppt using a bulk sensitivity of 4.8 ncps ppt^{-1} for all measured

ONs (see Section S2). No loss corrections have been applied to the data set. A CIMS using bromide as the reagent ion and coupled with a customized inlet (Albrecht et al., 2019) attached directly at the bottom of the chamber was also deployed (Wu et al., 2021).

Table S1. *Experimental conditions in the atmosphere simulation chamber SAPHIR.*

Conditions	Exp. 1 (9 August)	Exp. 2 (13 August)	Exp. 3 (16 August)	Exp. 4 (12 August)
Favored chemical regimes	RO_2 + HO_2	RO_2 enhanced	Isomerization enhanced	RO_2 enhanced
Roof	Closed	Closed	Closed + open	Closed + open
Seeds	No	No	Yes	No
Humidity	Dry	Dry	Wet	Dry
Isoprene / ppbv	3 / 3 / 1.5	6 / 8 / 7	2 / 1.5 / 2	2.5 / 2 / 2.5
O_3 / ppbv	100 / 47 / 28 / 32	105 / 20 / 12 / 37	100/ 30 / -	80 / 46 / 13
NO_2 / ppbv	5 / 3 / 3 / 2.5	23 / 12.5 / 7 / 13	4 / 2.5 / 2.5	12 / - / 1
Propene / ppbv	100 / 40	-	-	-
CO / ppmv	120	0	0.02	120

The yields of the total measured ONs, $C_4H_7NO_5$, and the sum of the major primary products $C_5H_9NO_5$ (hydroperoxide nitrates, INP), $C_5H_7NO_4$ (carbonyl nitrates, ICN), and $C_5H_9NO_4$ (hydroxy nitrates, IHN) were estimated based on isoprene consumption (Table S2). The measured ONs signal was converted to ppt using a bulk ON calibration factor of 4.8 ncps ppt^{-1} with a standard deviation of 0.7 ncps ppt^{-1} (Figure S1). The consumed isoprene was modeled and predicted based on measurements of isoprene, ozone, OH radicals, and dilution during the experiments. The OH concentration was below the detection limit during the experiments while the amount of isoprene reacted with NO_3 was calculated to be around 90% (Brownwood et al., 2021).

Table S2. Estimated yields of the total measured ONs, $C_4H_7NO_5$, and the sum of the major primary products ($C_5H_9NO_5$, $C_5H_7NO_4$, and $C_5H_9NO_4$). The yields were estimated when all the isoprene had been consumed. The yields in experiments 3 and 4 were calculated only for the dark period. An error of 30%, corresponding to an uncertainty of 2σ has been applied.

Experiment	Total measured ONs yield (%)	$C_4H_7NO_5$ yield (%)	Major C_5 yield (%)
1 (9 Aug)	108.8 ± 32.6	3.4± 1.0	41.6 ± 12.5
2 (13 Aug)	97.6 ± 29.3	9.7 ± 2.9	51.6 ± 15.5
3 (16 Aug)	32.4 ± 9.7	5.3 ± 1.6	13.1 ± 3.9
4 (12 Aug)	76.7 ± 23.0	7.6 ± 2.8	38.6 ± 11.6

Text S2. Sensitivity estimates

Recently, Zhang and Zhang (2021) showed that secondary chemistry processes in the ion molecule reaction (IMR) chamber of CIMS under high O_3 conditions can lead to adduct formation of organic molecules with IO_x^-, instead of making a cluster with I^-. This can lead to misinterpretations of the identified chemical formulas. However, the assignment of $C_4H_7NO_5$ formula as an adduct with iodide ($(I)C_4H_7NO_5^-$) and not as an adduct with IO_x^- ($(IO)C_4H_7NO_4^-$) are supported by the following three reasons.
A test experiment was conducted in the SAPHIR chamber in which pre-synthesized N_2O_5 was used as the source of NO_3 radicals. Thus, ozone was not present preventing formation of IO_x^-. The N_2O_5 was flowing continuously into the chamber while isoprene was injected four times. The chemical conditions of the experiment were in line with the experiments 2 and 4 (Figure 1b & 1d). In absence of ozone, a very similar formation rate of the $C_4H_7NO_5$ was observed. The relative contribution of $C_4H_7NO_5$ to the total measured ONs was slightly higher than 10%, similar to experiments 2 and 4. The test experiment is in support of the assignment as $(I)C_4H_7NO_5^-$. Furthermore, in another phase of the test experiment ozone was added after all isoprene had been consumed by NO_3 and MVK was also injected. The goal was to observe if MVK could produce $C_4H_7NO_5$ but the observations were too complicated due to additional secondary reaction and this experiment was not analyzed further. However, the addition of ozone caused an instant increase of the IO_x^- signals induced by ozone chemistry in the chemical ionization inlet. An instant signal increase, was observed for 8 minor ON products out of the total 64 identified ONs. For the target species $C_4H_7NO_5$ there were no indications of an instant increase but rather a much slower increase was observed which was attributed to further oxidation of primary products due to the additions of ozone and MVK. This test confirms very limited influence of potential IO_x^- effects on the targeted species described in this paper.
Secondly, similar results were observed during NO_3-initiated isoprene oxidation experiments in the Go:PAM flow reactor. In these experiments NO_3 radicals were produced only via the dissociation of synthesized N_2O_5. $C_4H_7NO_5$ formed with lower contribution as the residence time and the chemical conditions favored the primary products during these

experiments. Finally, the $C_4H_7NO_5$ formula was also observed by a Br-CIMS. The Br-CIMS $C_4H_7NO_5$ signal was showing a similar time profile as the I-CIMS signal.
The ion counts, i.e. normalized counts per second (ncps), derived for each species using the I-CIMS can be converted to concentration units using appropriate instrumental sensitivities, which can be derived from standards. Unfortunately, for the detected ONs there is a lack of standards making direct quantification difficult. However, two methods to derive limits on sensitivities were applied, i.e. using bulk sensitivity or relative sensitivity. These two methods constrain and strengthen the various conclusions of the overall and relative importance of the various organonitrates discussed in this work.
To derive bulk-sensitivities for the ONs a Thermal Dissociation Cavity Ring-down Spectrometer (TD-CRDS) was deployed during the experiments in the atmospheric simulation chamber SAPHIR (Brownwood et al., 2021). The comparison of the sum of ONs measured by the I-CIMS versus the total alkyl nitrates measured by the TD-CRDS provides an estimation of the bulk sensitivity for the ONs (Figure S1). The mean average bulk ON sensitivity of the four experiments was 4.8 ncps ppt^{-1} with a standard deviation of 0.7 ncps ppt^{-1}. This bulk sensitivity factor of 4.8 ncps ppt^{-1} was used to convert to concentrations all the data collected using the University of Gothenburg CIMS (GU-CIMS) in this study.

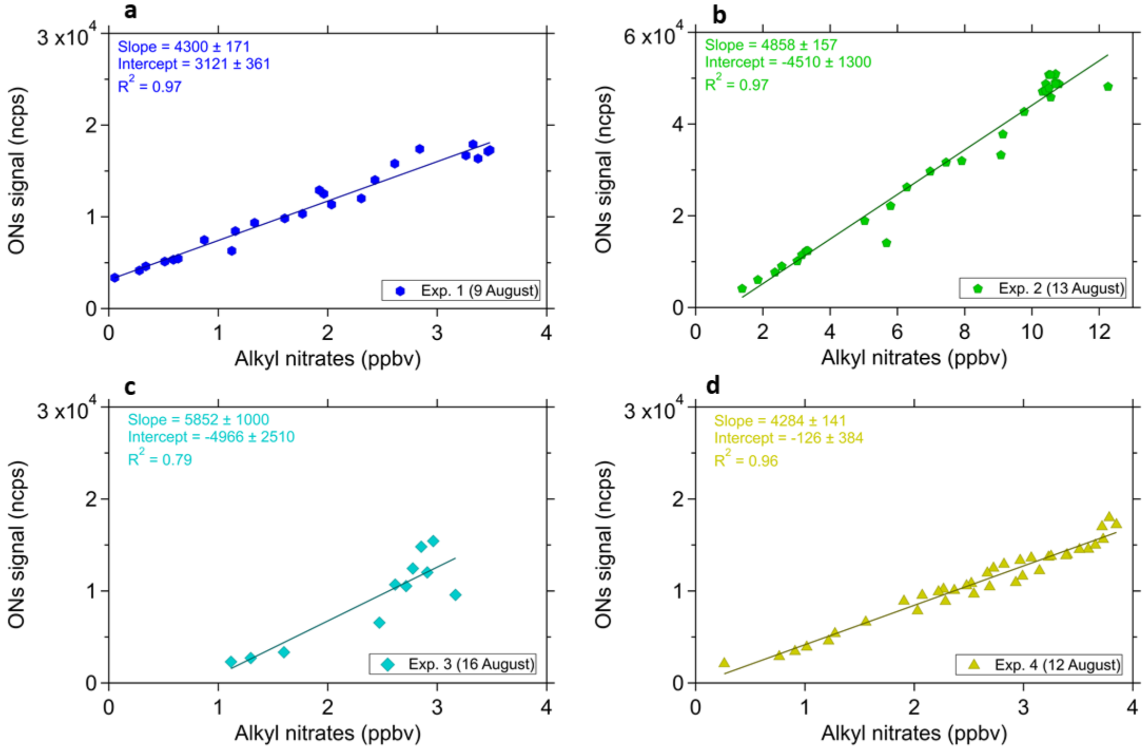

Figure S1. *Total ONs signal detected by I-CIMS versus total alkyl nitrates measured by the TD-CRDS.*

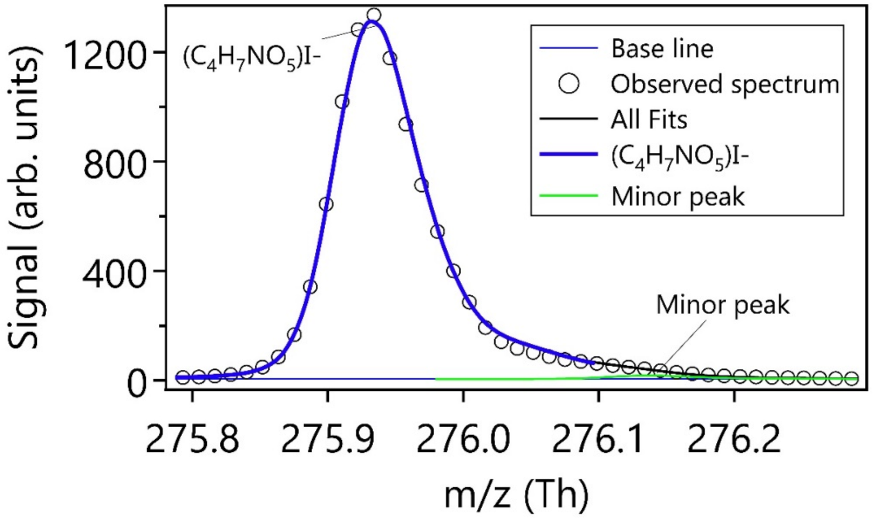

Figure S2. *An averaged spectrum at nominal mass-to-charge (m/z) 276 Th during the exp. 4 (Figure 1d). The $C_4H_7NO_5$ is the dominant peak at this nominal mass.*

In addition, a comparison with the $C_4H_7NO_5$ measurements using Br-CIMS are depicted in Figure S3. The comparison of normalized signal shows very good agreement between the two instruments, especially at the early oxidation stages, during experiments 1, 4 and the nighttime period of experiment 3. The discrepancy in experiment 3 after transition to the daytime might be attributed to different isomer distribution, because of OH oxidation. Experiment 2 is not shown since there were operation problems of the Br-CIMS that day.

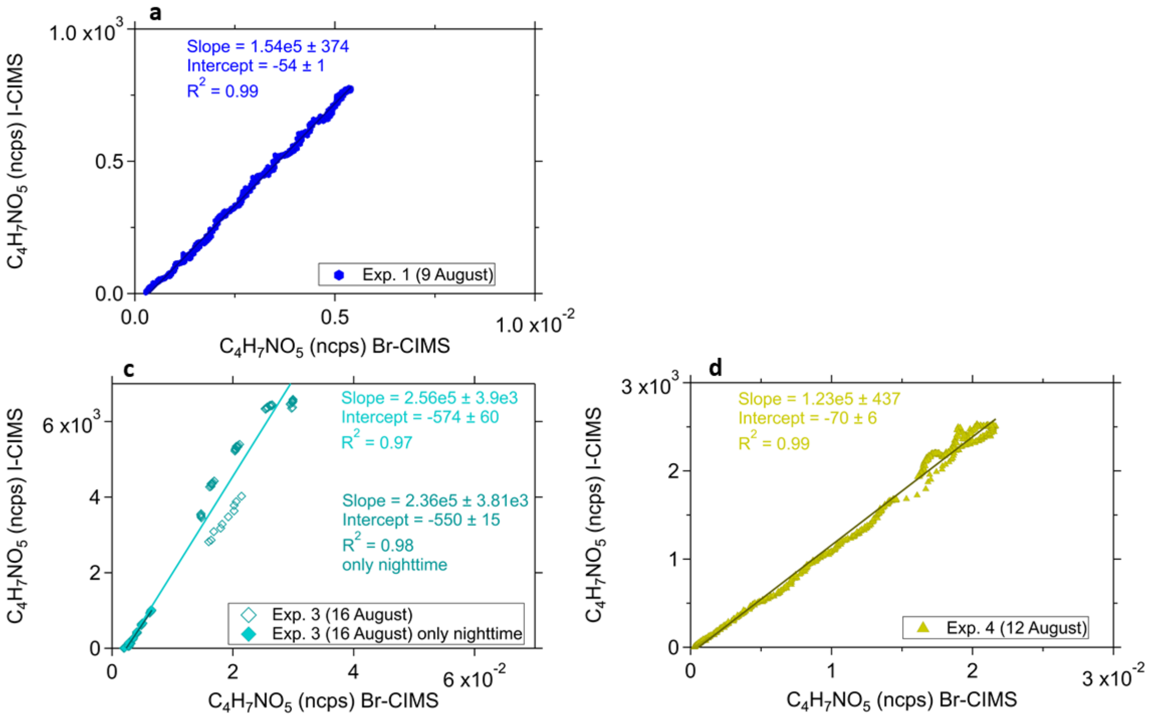

Figure S3. *Comparison of $C_4H_7NO_5$ measurements by I-CIMS and Br-CIMS.*

For differences in individual ON sensitivities the voltage scanning (VS) technique can be utilized to estimate an upper limit sensitivity. This will give a lower limit of concentration of the corresponding species, that is based on the collision limit of iodide ion adducts (Lopez-Hilfiker et al., 2016;Iyer et al., 2016). It should be noted that the I-CIMS can only provide the molecular formulas of the measured ions, which can consist of several different isomers. The iodide ionization can have isomer dependent sensitivities (e.g. IHN (Lee et al., 2016;Lee et al., 2014)) but this has not been considered here. During the VS technique the voltage difference (dV) of the interface between two quadrupoles in CIMS is increasing stepwise. The VS allows the determination of the voltage where half of the I-cluster signal is left (i.e dV_{50}). The dV_{50} value is then a measure of the stability of I-clusters which is an important property for its sensitivity.

We can estimate a relative sensitivity for different iodide adducts using a characterized compound concentration in conjunction with VS (Ye et al., 2021). We have characterized a dinitrogen pentoxide (N_2O_5) diffusion source via NO titration, and we obtained a calibration factor of 21 ncps ppt^{-1} which was used to determine the relative sensitivity. The N_2O_5 reacts with iodide ions at the collision limit and it can be used for the determination of the upper limit sensitivity (Lopez-Hilfiker et al., 2016). For the estimation of N_2O_5 the sum of NO_3^- (m/z 62) and $IN_2O_5^-$ (m/z 235) signals were used. Recently, Dörich et al. (2021) observed deprotonation of HNO_3 under high ozone mixing ratios by IO_x^- clusters in a similar mechanism to acetate CIMS measurements (Veres et al., 2008). This provides additional nitrate anions NO_3^- (m/z 62) that may lead to an overestimation of N_2O_5. However, in our work the calibration experiments and VS were done using pre-synthesized N_2O_5 in the absence of ozone, thus there was no formation of IO_x^- clusters that could deprotonate HNO_3. We performed oxidation experiments of isoprene and NO_3 using the Go:PAM flow reactor in conjunction with VS. The NO_3 radicals were introduced by dissociation of N_2O_5 via the characterized diffusion source. Then, we used the N_2O_5 calibration factor as the maximum sensitivity to estimate the relative sensitivity of products of interest.

It should be noted, that the VS experiments with the characterized N_2O_5 source were conducted after a general instrument refurbishment. This means that the instrument performance has been optimized compared to the period that the previous experiments took place (SAPHIR experiments and ambient measurements using the GU-CIMS). Thus, we expect higher sensitivities. However, we can derive useful information about the maximum sensitivity of specific products as well as any differences on their sensitivities.

The relative sensitivities of $C_4H_7NO_5$, $C_5H_9NO_5$ (INP), $C_5H_7NO_4$ (ICN), and $C_5H_9NO_4$ (IHN) were estimated using VS in five experiments. The average relative sensitivities and the standard deviations are given in the Table S3. The values of $C_4H_7NO_5$, $C_5H_9NO_5$, and $C_5H_7NO_4$ are similar indicating similar sensitivities, although the $C_5H_7NO_4$ value has higher uncertainty.

The $C_5H_9NO_4$ relative sensitivity is lower than the others but closer to values that has been reported before using synthesized standards of different $C_5H_9NO_4$ (IHN) isomers (Lee et al., 2014). This difference indicates that we may underestimate the $C_5H_9NO_4$ concentration. It should be noted that the relative sensitivities were estimated to understand the potential variability of I-CIMS sensitivity to the different ONs. The bulk ON calibration factor of 4.8

ncps ppt^{-1} with a standard deviation of 0.7 ncps ppt^{-1} was utilized for all conversions to ppt using GU-CIMS.

Table S3. *The estimated relative sensitivities of the ONs used for analysis based on the N_2O_5 calibration factor.*

	**Relative sensitivity (ncps ppt^{-1})**
**$C_4H_7NO_5$**	17.6 ± 1.7
**$C_5H_9NO_5$**	18.3 ± 2.9
**$C_5H_7NO_4$**	18.9 ± 6.7
**$C_5H_9NO_4$**	5.4 ± 3.4

Text S3. Ambient measurements

Data sets using an I-CIMS at six different locations around the globe have been used. University of Gothenburg CIMS (GU-CIMS) was used in Gothenburg, Sweden (2014), in Changping (near Beijing), China (2016), in Hong Kong (2018) and in Jülich, Germany (2019). The University of Washington instrument (UW-CIMS) was used in Centreville, Alabama, USA (2013). Finally, the University of Manchester CIMS (UMan-CIMS) was deployed in the Amazon rainforest, Brazil (2016).

The measured ONs signal has been converted using the bulk sensitivity factor for the ONs (4.8 ncps ppt^{-1}) at the locations where the GU-CIMS has been used. The measurements by the UW-CIMS used a weighted IHN ($C_5H_9NO_4$) isomer distribution as reported by Wennberg et al. (2018) and measured by Lee et al. (2014) as a proxy calibrant, while the UMan-CIMS used the isoprene-derived IEPOX as a proxy calibrant to convert the counts per seconds (cps) to ppt.

The measurements in Gothenburg, Sweden, took place in the city's port in October, 2014. The measurement in Changping and Hong Kong were part of the project "Photochemical smog in China" (Hallquist et al., 2016). The measurement site during the first campaign was located at a semi-rural area 40km north-east of downtown Beijing close to Changping town (Le Breton et al., 2018), while the second one was situated at the Hok Tsui Tsuen area, south-east on the Hong Kong island. The Changping measurements were conducted during May-June, 2016, while the Hong Kong ones during November-December, 2018. The measurements in Jülich took place in April-May, 2019 during the Jülich Atmospheric Chemistry Project campaign (JULIAC) at Forschungszentrum Jülich (FZJ), Germany. Here, ambient air was continuously sampled from an inlet at 50 m height above ground and then transferred to the atmospheric simulation chamber SAPHIR where all instruments sampled the air. The GU-CIMS was located in a container below SAPHIR.

The measurements in USA were part of the Southern Oxidant and Aerosol Study (SOAS) during June-July, 2013. The UW-CIMS was placed a few meters above the ground (Lee et al., 2016).

The Amazon campaign took place in June-July, 2016 in Central Amazonia, at a site located 60 km northwest of Manaus (0.235680° S, 60.12560° W, 110 m above sea level) facing a huge area (1600 km^2) of nearly pristine forest to the east. The UMan-CIMS was situated just above the canopy (~35 m height above ground).

Figure S4 shows the corresponding time-trends of the ON and NO_x shown as diurnal trends in Figure 2. The low NO_x levels observed could explain why isoprene-rich regions like Amazon and SE USA report relatively lower levels of selected ON. The ozone concentrations and operational characteristics of CIMS during each campaign are also depicted in Table S5 and Table S6, respectively.

Table S4. *Correlation slopes and R squared between $C_4H_7NO_5$ vs $C_5H_9NO_5$, $C_5H_7NO_4$, and $C_5H_9NO_4$ at the different measurement sites.*

		$C_4H_7NO_5$											
		Gothenburg		Amazon		Hong Kong		Changping		Jülich		Alabama	
		Cor. slope	R^2	Cor. slope	R^2	Cor. slope	R^2	Cor. slope	R^2	Cor. slope	R^2	Cor. slope	R^2
$C_5H_9NO_5$	Day	0.94	0.99	1.30	0.32	1.54	0.68	0.62	0.30	1.27	0.99	4.50	0.69
	Night	0.99	0.96	1.80	0.31	1.90	0.91	3.23	0.74	1.26	0.97	1.76	0.76
$C_5H_7NO_4$	Day	1.86	0.94	5.53	0.60	24.77	0.65	0.84	0.0006	6.29	0.33	151.98	0.31
	Night	1.90	0.80	4.26	0.12	19.87	0.57	16.42	0.25	2.57	0.07	21.58	0.37
$C_5H_9NO_4$	Day	-	-	3.19	0.74	9.99	0.88	-0.23	0.0015	6.95	0.45	4.49	0.78
	Night	-	-	4.07	0.38	6.81	0.70	2.97	0.07	7.60	0.50	3.63	0.92

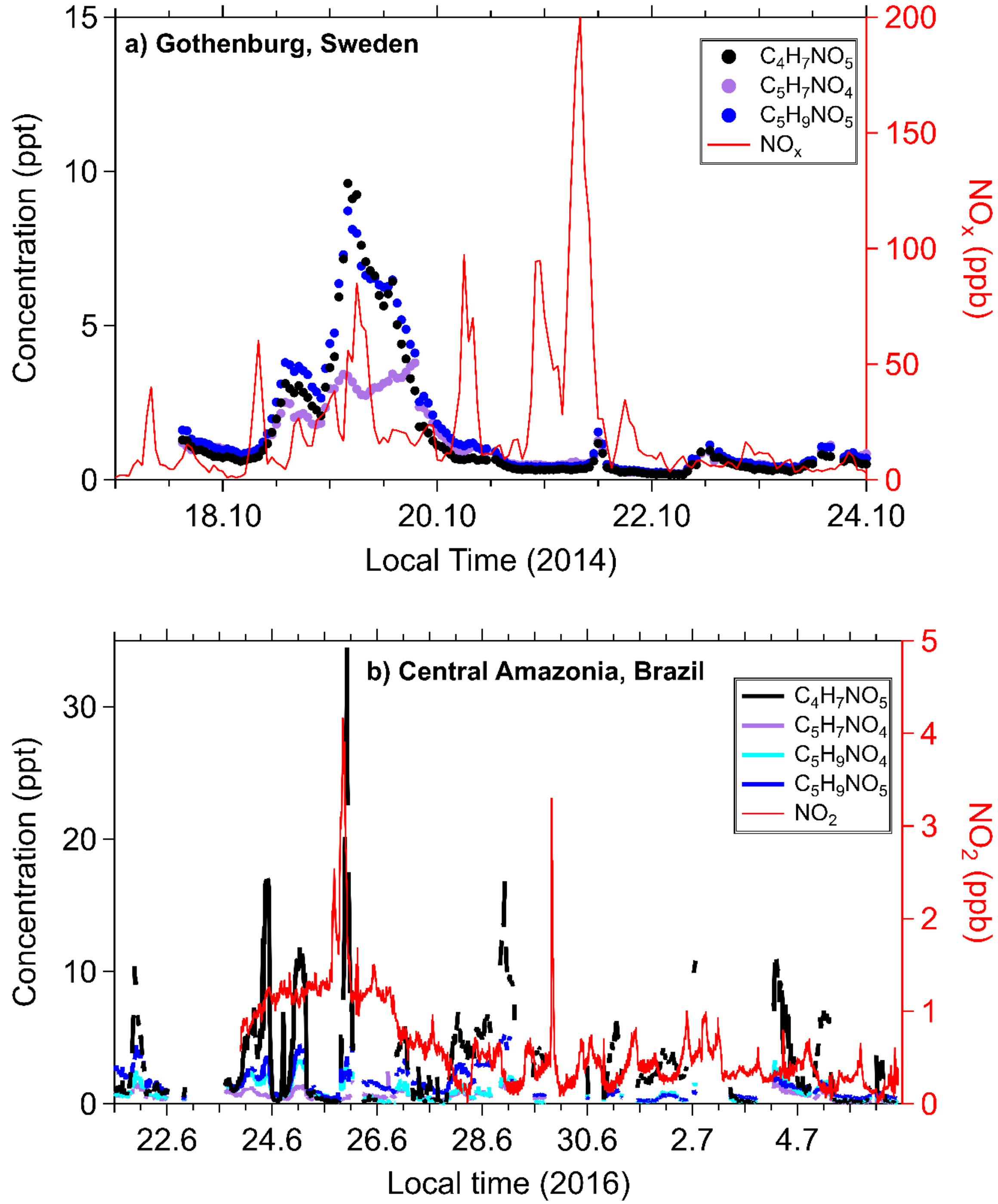
a) Gothenburg, Sweden
C4H7NO5
C5H7NO4
C5H9NO5
NOx
Concentration (ppt)
NOx (ppb)
0
5
10
15
0
50
100
150
200
18.10
20.10
22.10
24.10
Local Time (2014)
b) Central Amazonia, Brazil
C4H7NO5
C5H7NO4
C5H9NO4
C5H9NO5
NO2
Concentration (ppt)
NO2 (ppb)
0
10
20
30
0
1
2
3
4
5
22.6
24.6
26.6
28.6
30.6
2.7
4.7
Local time (2016)

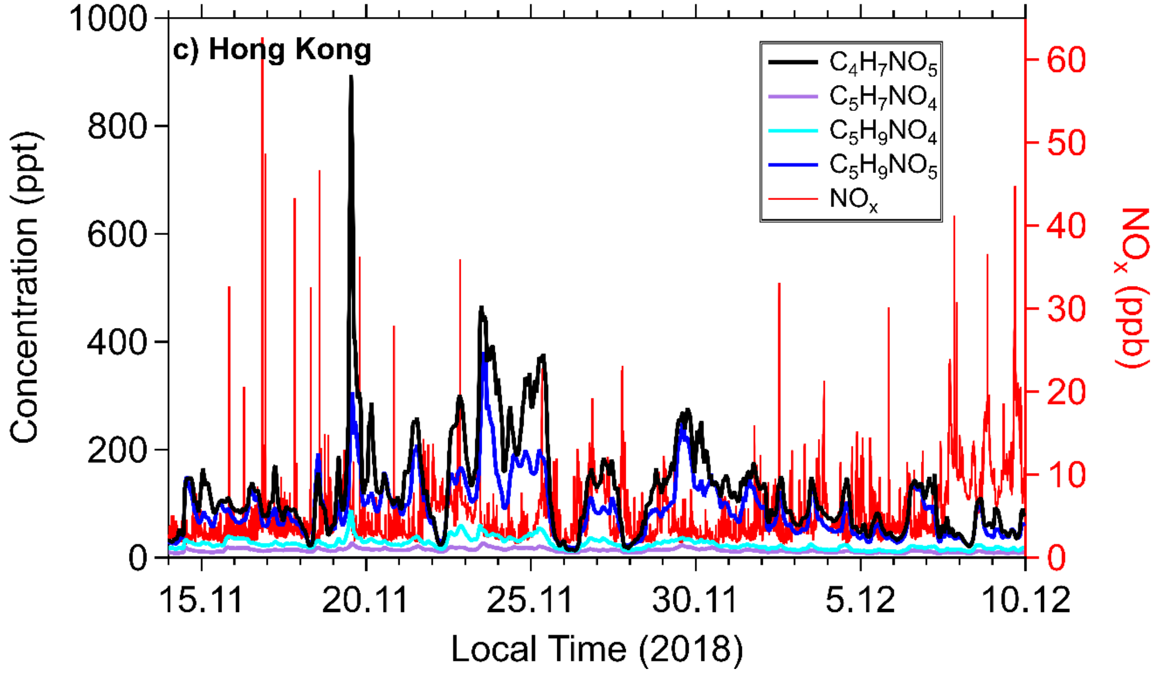
c) Hong Kong
$C_4H_7NO_5$
$C_5H_7NO_4$
$C_5H_9NO_4$
$C_5H_9NO_5$
NO_x
Concentration (ppt)
NO_x (ppb)
Local Time (2018)
15.11
20.11
25.11
30.11
5.12
10.12
0
200
400
600
800
1000
10
20
30
40
50
60

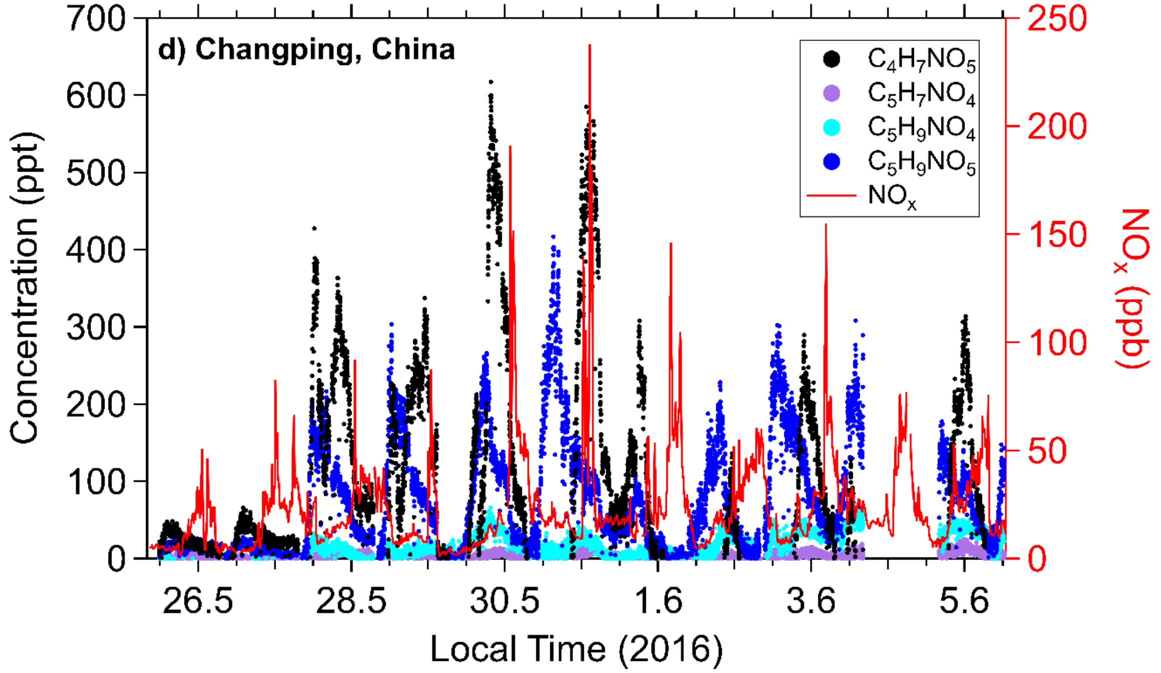
d) Changping, China
$C_4H_7NO_5$
$C_5H_7NO_4$
$C_5H_9NO_4$
$C_5H_9NO_5$
NO_x
Concentration (ppt)
NO_x (ppb)
Local Time (2016)
26.5
28.5
30.5
1.6
3.6
5.6
0
100
200
300
400
500
600
700
50
150
250

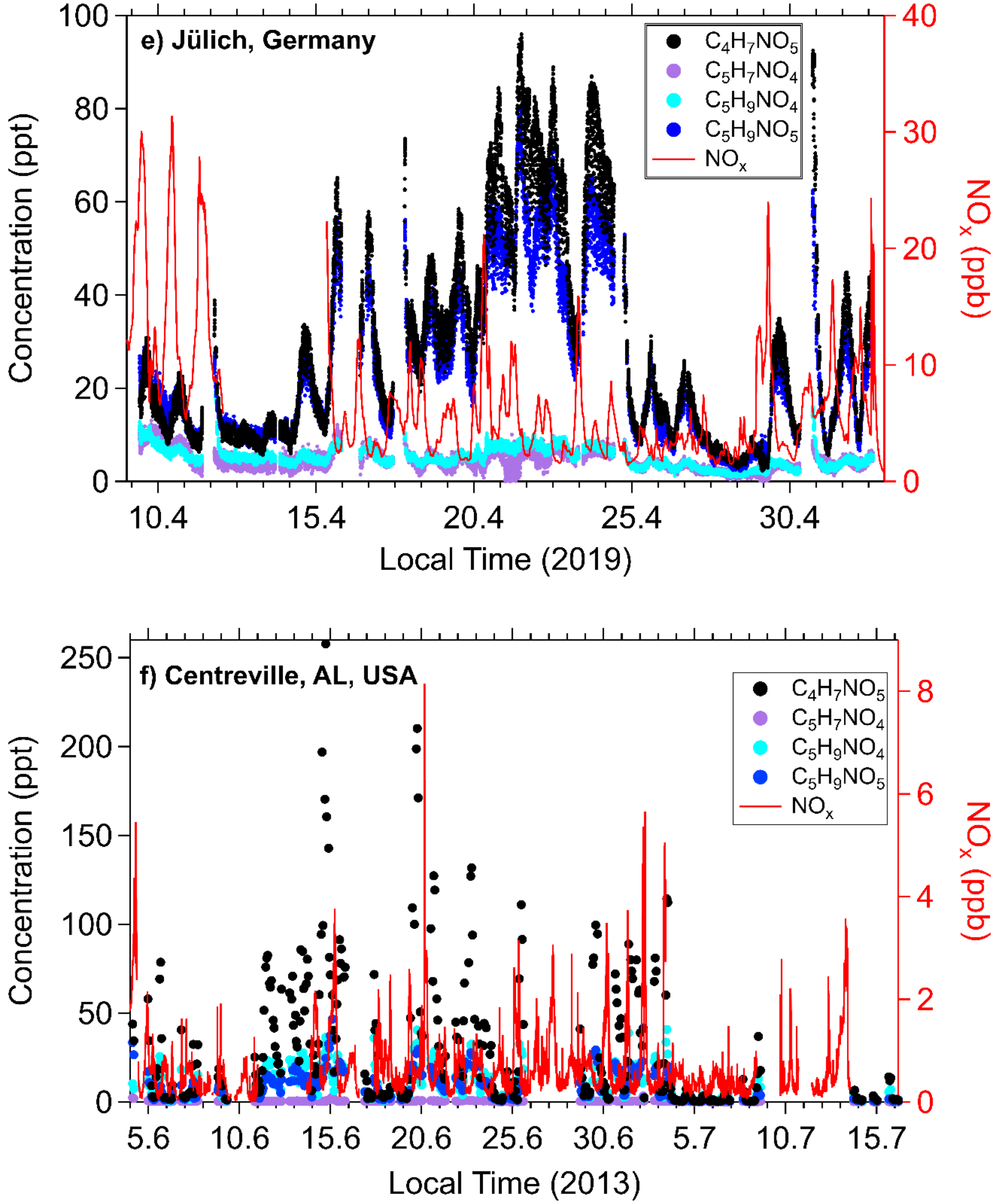

Figure S4. *The time series of the major isoprene nitrates and NO_x in a) Gothenburg, Sweden, b) Central Amazonia, Brazil, c) Hong Kong, d) Changping, China, e) Jülich, Germany, and f) Centreville, Alabama, USA. Note that only the NO_2 concentration is depicted in Amazonia.*

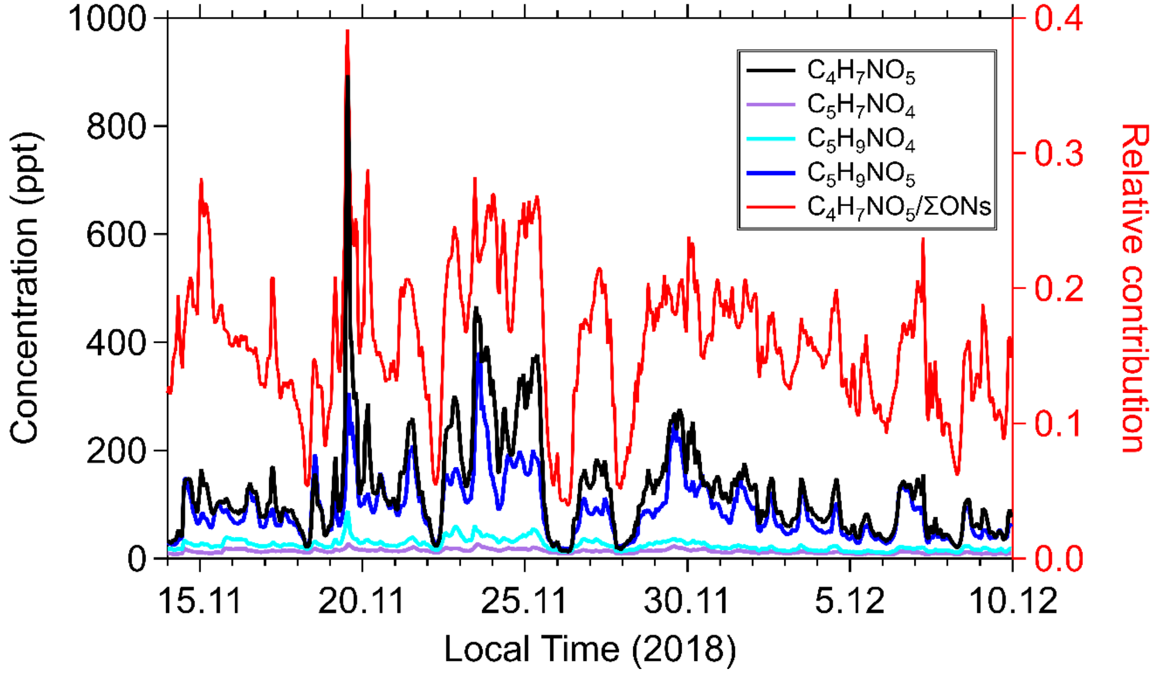

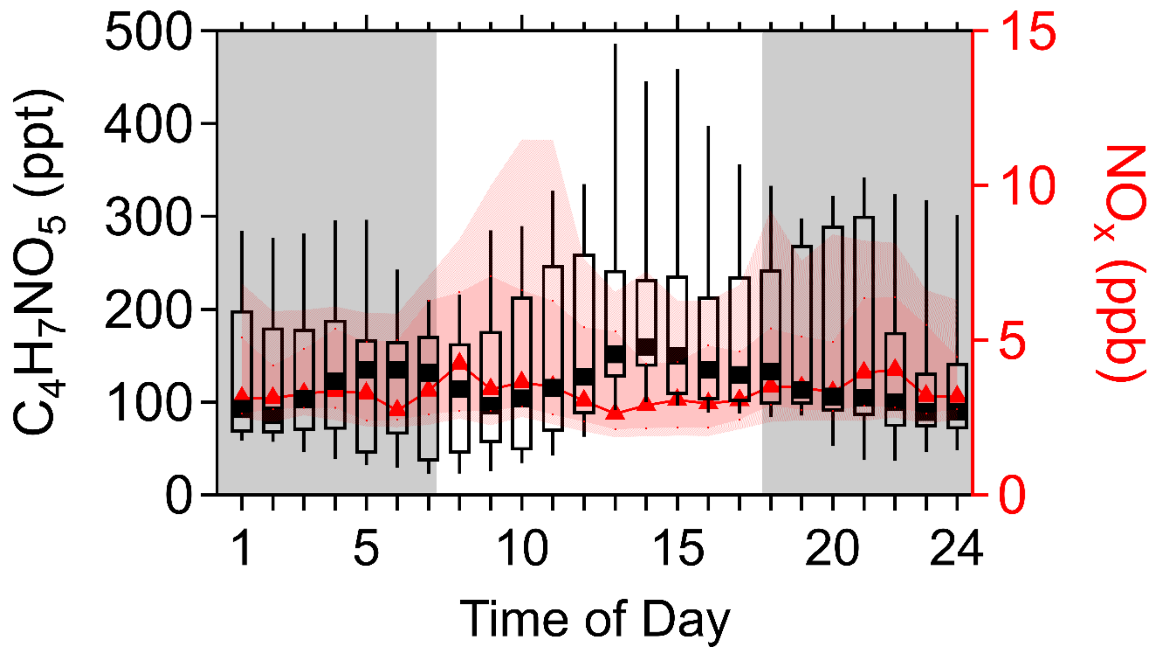

Figure S5. *(Top) The time series of the major isoprene nitrates in Hong Kong. The relative contribution represents the ratio of $C_4H_7NO_5$ over the total isoprene-derived nitrates that measured via the I-CIMS during the campaign. (Bottom) Median diurnal profile of the $C_4H_7NO_5$. and NO_x with the 10th, 25th, 75th and 90th percentile, in Hong Kong during the*

period with higher ONs formation (between 14-25/11). The grey areas in the plot indicate the nighttime period.

Table S5. *Mean, median, min and max ozone concentrations during the ambient observations at the different measurement sites.*

Ozone (ppb)	Gothenburg	Amazon	Hong Kong	Changping	Jülich	Alabama
Mean	22	12	53	58	41	27
Median	22	13	54	52	39	25
Min	2	0.1	3	0.6	6	0.3
Max	39	26	95	185	85	69

Table S6. *Operation characteristics of I-CIMS at the different measurement sites.*

	Gothenburg, GU-CIMS	Amazon, UMan-CIMS	Hong Kong, GU-CIMS	Changping, GU-CIMS	Jülich, GU-CIMS	Alabama, UW-CIMS
IMR[a] pressure (mbar)	190-199	-	147-248	418-441	165-220	-
SSQ[b] pressure (mbar)	1.79-1.80	-	0.85-1.0	1.77-1.78	1.05-1.1	-
Gas inflow (sccm)	~2000	~2000	~2000	~2000	~2050	~2000
Ionization	^{210}Po	^{210}Po	^{210}Po	X-ray	^{210}Po	^{210}Po

[a]IMR stands for ion molecule reaction. [b]SSQ stands for short segmented quadrupole.

Text S4. Chemical mechanism

The oxidation products with the chemical formula $C_4H_7NO_5$ consist of different isomers. The chemical structure of most relevant isomers and the naming convention using the Master Chemical Mechanism (MCM) are depicted in Table S7.

The FZJ-NO3-isoprene mechanism was published recently in the study by Vereecken et al. (2021). There, the focus was on the initial reaction of isoprene with the NO_3 radical and the resulting peroxy- and alkoxy-radicals. This also leads to additional sources of HC4ACHO and HC4CCHO (names as in the MCM, structures see Figures S5), for which plausible mechanisms for the formation of $C_4H_7NO_5$ can be formulated. With the help of structure activity relationships, the mechanisms shown in the Figure S5 were constructed. The rate of the reaction with NO_3 was calculated as shown in (Kerdouci et al., 2014), with a 65% preference for addition on the secondary carbon as used for the detailed description of the addition reaction in the MCM. The rate coefficients and branching ratios for the

bimolecular peroxy radical reaction pathways (reaction with NO, NO_3, HO_2 and other RO_2) are calculated according to (Jenkin et al., 2019), the unimolecular reactions as given in (Vereecken and Nozière, 2020). The rates for the alkoxy radicals are calculated as given in (Vereecken and Peeters, 2009;Novelli et al., 2021), with only the competitive reactions implemented. It should be noted that this is the first expansion of the FZJ-NO3-isoprene mechanism to such late-stage chemistry. The mechanism expansion itself with all relevant reactions are given below in EASY format:

```
k[HC4CCHO + NO3 --> ISOP1CO2OO3N4OH] = CONST(1.05E-14*0.65)
k[HC4CCHO + NO3 --> ISOP1CO2N3OO4OH]=CONST(1.05E-14*0.35)
k[HC4ACHO + NO3 --> ISOP1OH2OO3N4CO]=CONST(1.05E-14*0.65)
k[HC4ACHO + NO3 --> ISOP1OH2N3OO4CO]=CONST(1.05E-14*0.35)
k[ISOP1CO2OO3N4OH --> MVKNO3 + CO + OH] = CONST(2.54E-63*T@(24.25)*EXP(1605/T))
k[ISOP1CO2OO3N4OH + NO --> ISOP1CO2O3N4OH + NO2]=CONST(KRO2NO*0.93)
k[ISOP1CO2OO3N4OH + NO --> ISOP1CO2N3N4OH]=CONST(KRO2NO*0.07)
k[ISOP1CO2OO3N4OH + NO3 --> ISOP1CO2O3N4OH + NO2]=CONST(KRO2NO3)
k[ISOP1CO2OO3N4OH + HO2 --> ISOP1CO2OOH3N4OH]=CONST(2.58E-13*EXP(1300/T))
k[ISOP1CO2OO3N4OH --> ISOP1CO2OH3N4OH]=CONST(1.0E-13*EXP(-324/T)*0.3*RO2)
k[ISOP1CO2OO3N4OH --> ISOP1CO2O3N4OH]=CONST(1.0E-13*EXP(-324/T)*0.7*RO2)
k[ISOP1CO2N3OO4OH + NO --> ISOP1CO2N3O4OH + NO2]=CONST(KRO2NO*0.84)
k[ISOP1CO2N3OO4OH + NO --> ISOP1CO2N3N4OH]=CONST(KRO2NO*0.16)
k[ISOP1CO2N3OO4OH + NO3 --> ISOP1CO2N3O4OH + NO2]=CONST(KRO2NO3)
k[ISOP1CO2N3OO4OH + HO2 --> ISOP1CO2N3OOH4OH]=CONST(2.58E-13*EXP(1300/T))
k[ISOP1CO2N3OO4OH --> ISOP1CO2N3OH4OH]=CONST(1.0E-13*EXP(-790/T)*0.2*RO2)
k[ISOP1CO2N3OO4OH --> ISOP1CO2N3CO4OH]=CONST(1.0E-13*EXP(-790/T)*0.2*RO2)
k[ISOP1CO2N3OO4OH --> ISOP1CO2N3O4OH]=CONST(1.0E-13*EXP(-790/T)*0.6*RO2)
k[ISOP1OH2OO3N4CO + NO --> ISOP1OH2O3N4CO + NO2]=CONST(KRO2NO*0.84)
k[ISOP1OH2OO3N4CO + NO --> ISOP1OH2N3N4CO]=CONST(KRO2NO*0.16)
k[ISOP1OH2OO3N4CO + NO3 --> ISOP1OH2O3N4CO + NO2]=CONST(KRO2NO3)
k[ISOP1OH2OO3N4CO + HO2 --> ISOP1OH2OOH3N4CO]=CONST(2.58E-13*EXP(1300/T))
k[ISOP1OH2OO3N4CO --> ISOP1OH2OH3N4CO]=CONST(1.0E-13*EXP(-221/T)*0.3*RO2)
k[ISOP1OH2OO3N4CO --> ISOP1OH2O3N4CO]=CONST(1.0E-13*EXP(-221/T)*0.7*RO2)
k[ISOP1OH2N3OO4CO --> MACRNO3 + CO + OH] = CONST(1.08E-66*T@(25.23)*EXP(1616/T))
k[ISOP1OH2N3OO4CO + NO --> ISOP1OH2N3O4CO + NO2]=CONST(KRO2NO*0.93)
k[ISOP1OH2N3OO4CO + NO --> ISOP1OH2N3N4CO]=CONST(KRO2NO*0.07)
k[ISOP1OH2N3OO4CO + NO3 --> ISOP1OH2N3O4CO + NO2]=CONST(KRO2NO3)
k[ISOP1OH2N3OO4CO + HO2 --> ISOP1OH2N3OOH4CO]=CONST(2.58E-13*EXP(1300/T))
k[ISOP1OH2N3OO4CO --> ISOP1OH2N3OH4CO]=CONST(1.0E-13*EXP(-893/T)*0.2*RO2)
k[ISOP1OH2N3OO4CO --> ISOP1OH2N3CO4CO]=CONST(1.0E-13*EXP(-893/T)*0.2*RO2)
k[ISOP1OH2N3OO4CO --> ISOP1OH2N3O4CO]=CONST(1.0E-13*EXP(-893/T)*0.6*RO2)
k[ISOP1CO2O3N4OH --> MGLYOX + HOCH2CHO + NO2] = CONST(1.8E13*(T/298)@(1.7)*EXP(-4630/T))
k[ISOP1CO2O3N4OH --> MVKNO3 + CO + HO2] = CONST(1.8E13*(T/298)@(1.7)*EXP(-2567/T))
k[ISOP1CO2N3O4OH --> CONM2CHO + HCHO + HO2]=CONST(1.8E13*(T/298)@(1.7)*EXP(-4076/T))
k[ISOP1CO2N3O4OH --> MGLYOX + HOCH2CHO + NO2]=CONST(1.8E13*(T/298)@(1.7)*EXP(-5032/T))
k[ISOP1CO2N3O4OH + O2 --> HO2 + ISOP1CO2N3CO4OH] = CONST(2.5E-14*EXP(-300./T))
k[ISOP1OH2O3N4CO --> ACETOL + GLYOX + NO2]=CONST(1.8E13*(T/298)@(1.7)*EXP(-4630/T))
k[ISOP1OH2O3N4CO --> C4CONO3CO + HCHO + HO2]=CONST(1.8E13*(T/298)@(1.7)*EXP(-2978/T))
k[ISOP1OH2N3O4CO --> ACETOL + GLYOX + NO2]=CONST(1.8E13*(T/298)@(1.7)*EXP(-5032/T))
k[ISOP1OH2N3O4CO --> MACRNO3 + CO + HO2]=CONST(1.8E13*(T/298)@(1.7)*EXP(-3573/T))
k[ISOP1OH2N3O4CO + O2 --> HO2 + ISOP1OH2N3CO4CO] = CONST(2.5E-14*EXP(-300./T))
```

Table S7. *Molecular structure of the different isomers with chemical formula* $C_4H_7NO_5$ *and their corresponding MCM name.*

Molecular Structure	MCM name
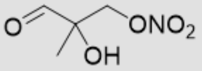	MACRNB
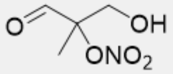	MACRNO3
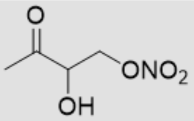	HMVKANO3
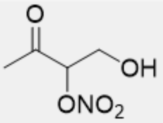	MVKNO3

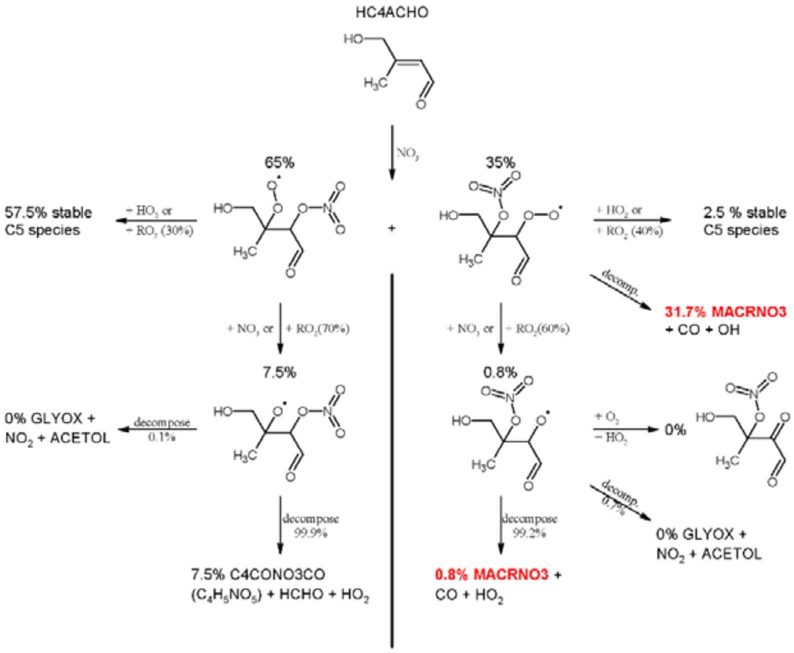

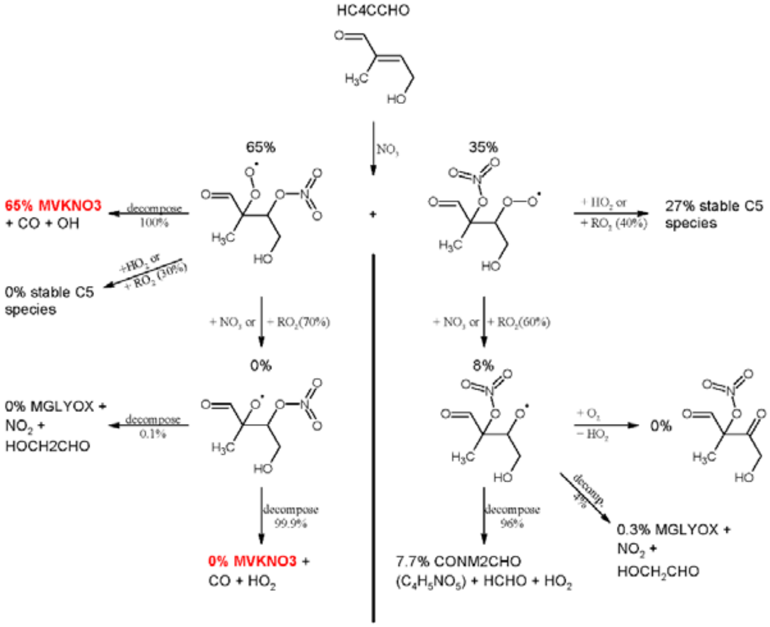

Figure S6. *(Top) Analytical formation pathways of $C_4H_7NO_5$ isomer by HC4CCHO (reaction R6 in the main text). (Bottom) Analytical formation pathways of $C_4H_7NO_5$ isomer by HC4ACHO (reaction R7 in the main text).*

The comparison of the modeled and the measured $C_4H_7NO_5$ time profiles are in good agreement for experiments 2 and 3 (Figure S7 and S8) (see also Figure 3 and Table S1). However, the estimated concentrations differ between the model and the measurements. The discrepancy varies depending on the experiment by a factor of 10 to 22. The lowest difference was observed for experiment 2 (a factor of 10). One may note that in experiment 1 there was addition of propene. The model has not been utilized for the additional propene chemistry, thus there is higher discrepancy for experiment 1 compared to the others.

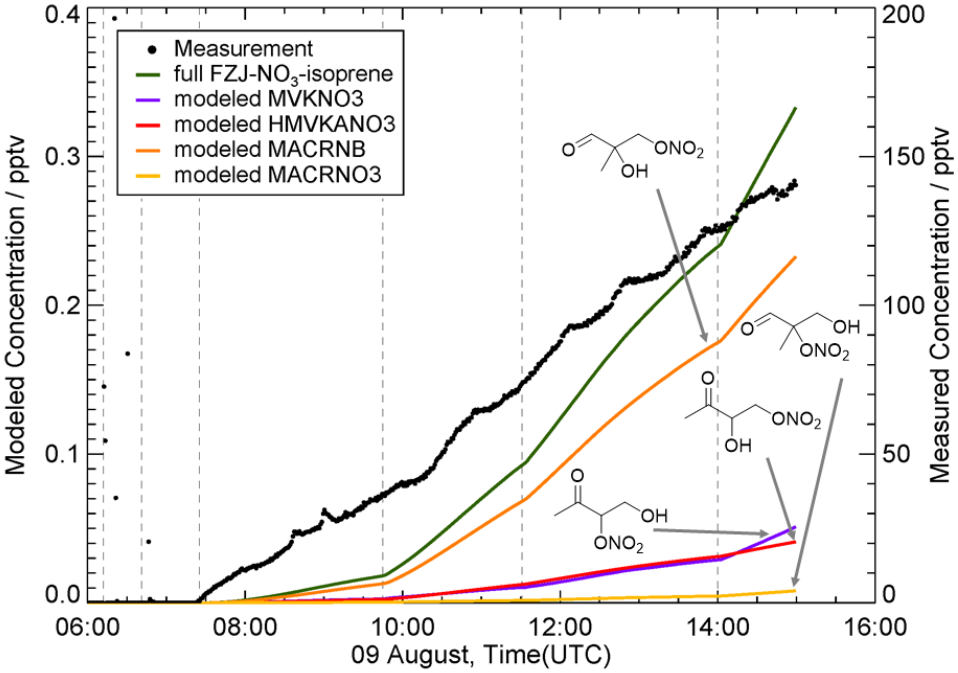

Figure S7. *Comparison of the measured (black) and modeled (green) $C_4H_7NO_5$ formation during exp. 1 (enhanced RO_2 + HO_2, addition of propene). The sum "full FZJ-NO_3-isoprene" of the four main isomers is compared against the I-CIMS measurements.*

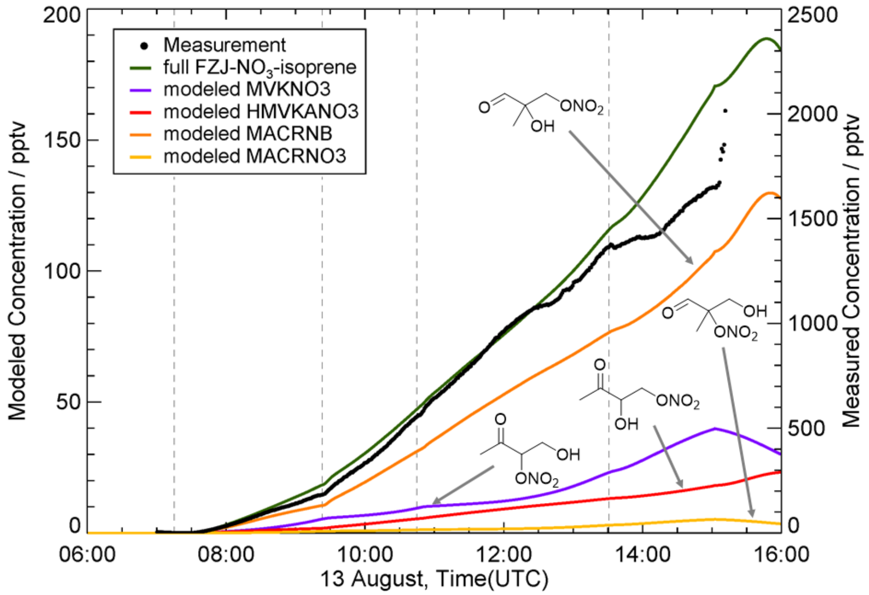

Figure S8. *Comparison of the measured (black) and modeled (green) $C_4H_7NO_5$ formation during exp. 2 (enhanced RO_2). The sum "full FZJ-NO_3-isoprene" of the four main isomers is compared against the I-CIMS measurements.*

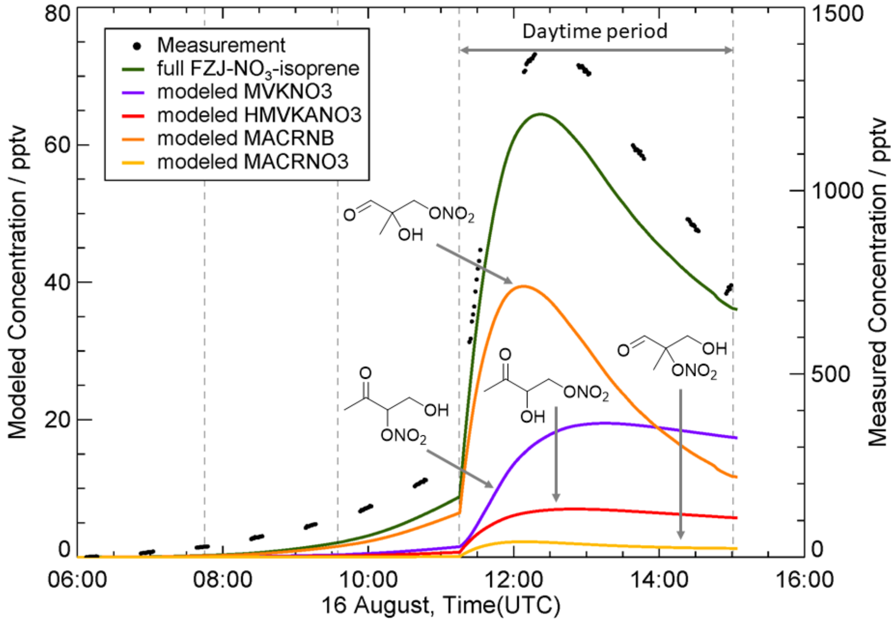

Figure S9. *Comparison of the measured (black) and modeled (green) $C_4H_7NO_5$ formation during exp. 3 (nighttime to daytime transition). The sum "full FZJ-NO_3-isoprene" of the four main isomers is compared against the I-CIMS measurements.*

Text S5. Go:PAM experiments

Oxidation experiments of MVK by NO_3 radicals were conducted using the laminar-flow Go:PAM reactor (Tsiligiannis et al., 2019;Watne et al., 2018) and the I-CIMS. The NO_3 radicals were introduced by dissociation of N_2O_5 via a continuous diffusion source. N_2O_5 was synthesized by reacting pure NO_2 with excess ozone in a glass vessel. The formed N_2O_5 was collected passing through a cold trap (-78.5 °C) using dry ice and then transferred to a diffusion vial fitted with a capillary tube (Faxon et al., 2018). The transfer took place in a dry glove box to minimize the exposure to ambient moisture. The N_2O_5 diffusion source was kept in a water bath at a stable temperature, which was varied to control NO_3 concentration. The sum of the signals of NO_3^- product at m/z 62 and the adduct ion of $IN_2O_5^-$ at m/z 235 was used to estimate the NO_3 concentration. The NO_3 was quantified introducing pure NO to titrate the NO_3 to NO_2 while it was monitored by a NO/NO_x analyzer (CLD 700 AL chemiluminescence).
MVK was introduced via a second diffusion vial fitted with a capillary tube and held in a water bath to control the temperature. Here the diffusion flux was determined gravimetrically. Using the NO_3 loss due to MVK reaction and the produced amount of $C_4H_7NO_5$ as measured by the I-CIMS, an upper limit of the primary yield could be estimated to less than 5 ncps per ppb of NO_3 consumed (i.e., less than 0.01%) (Figure S10).

Table S8. *Experimental conditions of MVK oxidation by NO_3 radicals in the Go:PAM flow reactor.*

	$[MVK]_0$ (ppb)	$[NO_3+N_2O_5]_0$ (ppb)	$\Delta[NO_3+N_2O_5]$ (ppb)	$C_4H_7NO_5$ production (ncps)
1	200	6	0.57	3.69
2	200	30	1.26	11.87
3	200	83	2.60	17.87
4	500	6	3.34	4.63
5	500	27	7.41	27.20
6	500	30	8.07	28.84
7	500	92	11.80	53.19

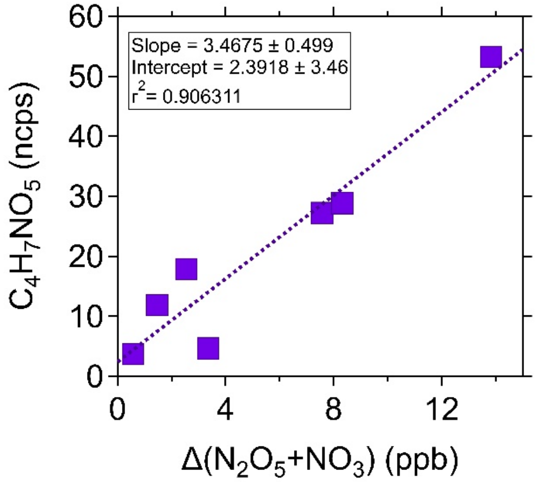

Figure S10. *$C_4H_7NO_5$ formation versus the consumed oxidant during the oxidation of MVK by NO_3 radicals in the Go:PAM flow reactor. NO_3 was introduced using a N_2O_5 diffusion source with subsequent decomposition.*

References

Albrecht, S. R., Novelli, A., Hofzumahaus, A., Kang, S., Baker, Y., Mentel, T., Wahner, A., and Fuchs, H.: Measurements of hydroperoxy radicals (HO2) at atmospheric concentrations using bromide chemical ionisation mass spectrometry, Atmos. Meas. Tech., 12, 891-902, 10.5194/amt-12-891-2019, 2019.

Brownwood, B., Turdziladze, A., Hohaus, T., Wu, R., Mentel, T. F., Carlsson, P. T. M., Tsiligiannis, E., Hallquist, M., Andres, S., Hantschke, L., Reimer, D., Rohrer, F., Tillmann, R., Winter, B., Liebmann, J., Brown, S. S., Kiendler-Scharr, A., Novelli, A., Fuchs, H., and Fry, J. L.: Gas-Particle Partitioning and SOA Yields of Organonitrate Products from NO3-Initiated Oxidation of Isoprene under Varied Chemical Regimes, ACS Earth and Space Chemistry, 10.1021/acsearthspacechem.0c00311, 2021.

Dewald, P., Liebmann, J. M., Friedrich, N., Shenolikar, J., Schuladen, J., Rohrer, F., Reimer, D., Tillmann, R., Novelli, A., Cho, C., Xu, K., Holzinger, R., Bernard, F., Zhou, L., Mellouki, W., Brown, S. S., Fuchs, H., Lelieveld, J., and Crowley, J. N.: Evolution of NO3 reactivity during the oxidation of isoprene, Atmos. Chem. Phys., 20, 10459-10475, 10.5194/acp-20-10459-2020, 2020.

Dörich, R., Eger, P., Lelieveld, J., and Crowley, J. N.: Iodide CIMS and m⁄z 62: the detection of HNO3 as NO3− in the presence of PAN, peroxyacetic acid and ozone, Atmos. Meas. Tech., 14, 5319-5332, 10.5194/amt-14-5319-2021, 2021.

Faxon, C., Hammes, J., Le Breton, M., Pathak, R. K., and Hallquist, M.: Characterization of organic nitrate constituents of secondary organic aerosol (SOA) from nitrate-radical-initiated oxidation of limonene using high-resolution chemical ionization mass spectrometry, Atmos. Chem. Phys., 18, 5467-5481, 10.5194/acp-18-5467-2018, 2018.

Fuchs, H., Novelli, A., Rolletter, M., Hofzumahaus, A., Pfannerstill, E. Y., Kessel, S., Edtbauer, A., Williams, J., Michoud, V., Dusanter, S., Locoge, N., Zannoni, N., Gros, V., Truong, F., Sarda-Esteve, R., Cryer, D. R., Brumby, C. A., Whalley, L. K., Stone, D., Seakins, P. W., Heard, D. E., Schoemaecker, C., Blocquet, M., Coudert, S., Batut, S.,

Fittschen, C., Thames, A. B., Brune, W. H., Ernest, C., Harder, H., Muller, J. B. A., Elste, T., Kubistin, D., Andres, S., Bohn, B., Hohaus, T., Holland, F., Li, X., Rohrer, F., Kiendler-Scharr, A., Tillmann, R., Wegener, R., Yu, Z., Zou, Q., and Wahner, A.: Comparison of OH reactivity measurements in the atmospheric simulation chamber SAPHIR, Atmos. Meas. Tech., 10, 4023-4053, 10.5194/amt-10-4023-2017, 2017.

Hallquist, M., Munthe, J., Hu, M., Wang, T., Chan, C. K., Gao, J., Boman, J., Guo, S., Hallquist, Å. M., Mellqvist, J., Moldanova, J., Pathak, R. K., Pettersson, J. B., Pleijel, H., Simpson, D., and Thynell, M.: Photochemical smog in China: scientific challenges and implications for air-quality policies, National Science Review, 3, 401-403, 10.1093/nsr/nww080, 2016.

Iyer, S., Lopez-Hilfiker, F., Lee, B. H., Thornton, J. A., and Kurten, T.: Modeling the Detection of Organic and Inorganic Compounds Using Iodide-Based Chemical Ionization, J Phys Chem A, 120, 576-587, 10.1021/acs.jpca.5b09837, 2016.

Jenkin, M. E., Valorso, R., Aumont, B., and Rickard, A. R.: Estimation of rate coefficients and branching ratios for reactions of organic peroxy radicals for use in automated mechanism construction, Atmos. Chem. Phys., 19, 7691-7717, 10.5194/acp-19-7691-2019, 2019.

Kerdouci, J., Picquet-Varrault, B., and Doussin, J.-F.: Structure–activity relationship for the gas-phase reactions of NO3 radical with organic compounds: Update and extension to aldehydes, Atmospheric Environment, 84, 363-372, https://doi.org/10.1016/j.atmosenv.2013.11.024, 2014.

Le Breton, M., Wang, Y., Hallquist, Å. M., Pathak, R. K., Zheng, J., Yang, Y., Shang, D., Glasius, M., Bannan, T. J., Liu, Q., Chan, C. K., Percival, C. J., Zhu, W., Lou, S., Topping, D., Wang, Y., Yu, J., Lu, K., Guo, S., Hu, M., and Hallquist, M.: Online gas- and particle-phase measurements of organosulfates, organosulfonates and nitrooxy organosulfates in Beijing utilizing a FIGAERO ToF-CIMS, Atmos. Chem. Phys., 18, 10355-10371, 10.5194/acp-18-10355-2018, 2018.

Lee, B. H., Lopez-Hilfiker, F. D., Mohr, C., Kurten, T., Worsnop, D. R., and Thornton, J. A.: An iodide-adduct high-resolution time-of-flight chemical-ionization mass spectrometer: application to atmospheric inorganic and organic compounds, Environ Sci Technol, 48, 6309-6317, 10.1021/es500362a, 2014.

Lee, B. H., Mohr, C., Lopez-Hilfiker, F. D., Lutz, A., Hallquist, M., Lee, L., Romer, P., Cohen, R. C., Iyer, S., Kurten, T., Hu, W., Day, D. A., Campuzano-Jost, P., Jimenez, J. L., Xu, L., Ng, N. L., Guo, H., Weber, R. J., Wild, R. J., Brown, S. S., Koss, A., de Gouw, J., Olson, K., Goldstein, A. H., Seco, R., Kim, S., McAvey, K., Shepson, P. B., Starn, T., Baumann, K., Edgerton, E. S., Liu, J., Shilling, J. E., Miller, D. O., Brune, W., Schobesberger, S., D'Ambro, E. L., and Thornton, J. A.: Highly functionalized organic nitrates in the southeast United States: Contribution to secondary organic aerosol and reactive nitrogen budgets, Proc Natl Acad Sci U S A, 113, 1516-1521, 10.1073/pnas.1508108113, 2016.

Lopez-Hilfiker, F. D., Mohr, C., Ehn, M., Rubach, F., Kleist, E., Wildt, J., Mentel, T. F., Lutz, A., Hallquist, M., Worsnop, D., and Thornton, J. A.: A novel method for online analysis of gas and particle composition: description and evaluation of a Filter Inlet for Gases and AEROsols (FIGAERO), Atmospheric Measurement Techniques, 7, 983-1001, 10.5194/amt-7-983-2014, 2014.

Lopez-Hilfiker, F. D., Iyer, S., Mohr, C., Lee, B. H., D'Ambro, E. L., Kurtén, T., and Thornton, J. A.: Constraining the sensitivity of iodide adduct chemical ionization mass spectrometry to multifunctional organic molecules using the collision limit and thermodynamic stability of iodide ion adducts, Atmos. Meas. Tech., 9, 1505-1512, 10.5194/amt-9-1505-2016, 2016.

Novelli, A., Cho, C., Fuchs, H., Hofzumahaus, A., Rohrer, F., Tillmann, R., Kiendler-Scharr, A., Wahner, A., and Vereecken, L.: Experimental and theoretical study on the impact of a nitrate group on the chemistry of alkoxy radicals, Physical Chemistry Chemical Physics, 10.1039/D0CP05555G, 2021.

Rohrer, F., Bohn, B., Brauers, T., Brüning, D., Johnen, F. J., Wahner, A., and Kleffmann, J.: Characterisation of the photolytic HONO-source in the atmosphere simulation chamber SAPHIR, Atmos. Chem. Phys., 5, 2189-2201, 10.5194/acp-5-2189-2005, 2005.

Tsiligiannis, E., Hammes, J., Salvador, C. M., Mentel, T. F., and Hallquist, M.: Effect of NOx on 1,3,5-trimethylbenzene (TMB) oxidation product distribution and particle formation, Atmos. Chem. Phys., 19, 15073-15086, 10.5194/acp-19-15073-2019, 2019.

Watne, A. K., Psichoudaki, M., Ljungstrom, E., Le Breton, M., Hallquist, M., Jerksjo, M., Fallgren, H., Jutterstrom, S., and Hallquist, A. M.: Fresh and Oxidized Emissions from In-Use Transit Buses Running on Diesel, Biodiesel, and CNG, Environ Sci Technol, 52, 7720-7728, 10.1021/acs.est.8b01394, 2018.

Wennberg, P. O., Bates, K. H., Crounse, J. D., Dodson, L. G., McVay, R. C., Mertens, L. A., Nguyen, T. B., Praske, E., Schwantes, R. H., Smarte, M. D., St Clair, J. M., Teng, A. P., Zhang, X., and Seinfeld, J. H.: Gas-Phase Reactions of Isoprene and Its Major Oxidation Products, Chemical Reviews, 118, 3337-3390, 10.1021/acs.chemrev.7b00439, 2018.

Vereecken, L., and Peeters, J.: Decomposition of substituted alkoxy radicals—part I: a generalized structure–activity relationship for reaction barrier heights, Physical Chemistry Chemical Physics, 11, 9062-9074, 10.1039/B909712K, 2009.

Vereecken, L., and Nozière, B.: H migration in peroxy radicals under atmospheric conditions, Atmos. Chem. Phys., 20, 7429-7458, 10.5194/acp-20-7429-2020, 2020.

Vereecken, L., Carlsson, P. T. M., Novelli, A., Bernard, F., Brown, S. S., Cho, C., Crowley, J. N., Fuchs, H., Mellouki, W., Reimer, D., Shenolikar, J., Tillmann, R., Zhou, L., Kiendler-Scharr, A., and Wahner, A.: Theoretical and experimental study of peroxy and alkoxy radicals in the NO3-initiated oxidation of isoprene, Physical Chemistry Chemical Physics, 10.1039/D0CP06267G, 2021.

Veres, P., Roberts, J. M., Warneke, C., Welsh-Bon, D., Zahniser, M., Herndon, S., Fall, R., and de Gouw, J.: Development of negative-ion proton-transfer chemical-ionization mass spectrometry (NI-PT-CIMS) for the measurement of gas-phase organic acids in the atmosphere, Int J Mass Spectrom, 274, 48-55, 10.1016/j.ijms.2008.04.032, 2008.

Wu, R., Vereecken, L., Tsiligiannis, E., Kang, S., Albrecht, S. R., Hantschke, L., Zhao, D., Novelli, A., Fuchs, H., Tillmann, R., Hohaus, T., Carlsson, P. T. M., Shenolikar, J., Bernard, F., Crowley, J. N., Fry, J. L., Brownwood, B., Thornton, J. A., Brown, S. S., Kiendler-Scharr, A., Wahner, A., Hallquist, M., and Mentel, T. F.: Molecular composition and volatility of multi-generation products formed from isoprene oxidation by nitrate radical, Atmos. Chem. Phys., 21, 10799-10824, 10.5194/acp-21-10799-2021, 2021.

Ye, C., Yuan, B., Lin, Y., Wang, Z., Hu, W., Li, T., Chen, W., Wu, C., Wang, C., Huang, S., Qi, J., Wang, B., Wang, C., Song, W., Wang, X., Zheng, E., Krechmer, J. E., Ye, P., Zhang, Z., Wang, X., Worsnop, D. R., and Shao, M.: Chemical characterization of oxygenated organic compounds in the gas phase and particle phase using iodide CIMS with FIGAERO in urban air, Atmos. Chem. Phys., 21, 8455-8478, 10.5194/acp-21-8455-2021, 2021.
Zhang, W., and Zhang, H.: Secondary Ion Chemistry Mediated by Ozone and Acidic Organic Molecules in Iodide-Adduct Chemical Ionization Mass Spectrometry, Analytical chemistry, 93, 8595-8602, 10.1021/acs.analchem.1c01486, 2021.
